# Patterning two-dimensional chalcogenide crystals of Bi_2_Se_3_ and In_2_Se_3_ and efficient photodetectors

**DOI:** 10.1038/ncomms7972

**Published:** 2015-04-21

**Authors:** Wenshan Zheng, Tian Xie, Yu Zhou, Y.L. Chen, Wei Jiang, Shuli Zhao, Jinxiong Wu, Yumei Jing, Yue Wu, Guanchu Chen, Yunfan Guo, Jianbo Yin, Shaoyun Huang, H.Q. Xu, Zhongfan Liu, Hailin Peng

**Affiliations:** 1 Center for Nanochemistry, Beijing National Laboratory for Molecular Sciences (BNLMS), State Key Laboratory for Structural Chemistry of Unstable and Stable Species, College of Chemistry and Molecular Engineering, Peking University, Beijing 100871, P. R. China; 2 Department of Physics and Clarendon Laboratory, University of Oxford, Parks Road, Oxford OX1 3PU, UK; 3 Key Laboratory for the Physics and Chemistry of Nanodevices, Department of Electronics, Peking University, Beijing 100871, P. R. China; 4 Division of Solid State Physics, Lund University, Box 118, S-221 00 Lund, Sweden

## Abstract

Patterning of high-quality two-dimensional chalcogenide crystals with unique planar structures and various fascinating electronic properties offers great potential for batch fabrication and integration of electronic and optoelectronic devices. However, it remains a challenge that requires accurate control of the crystallization, thickness, position, orientation and layout. Here we develop a method that combines microintaglio printing with van der Waals epitaxy to efficiently pattern various single-crystal two-dimensional chalcogenides onto transparent insulating mica substrates. Using this approach, we have patterned large-area arrays of two-dimensional single-crystal Bi_2_Se_3_ topological insulator with a record high Hall mobility of ∼1,750 cm^2^ V^−1^ s^−1^ at room temperature. Furthermore, our patterned two-dimensional In_2_Se_3_ crystal arrays have been integrated and packaged to flexible photodetectors, yielding an ultrahigh external photoresponsivity of ∼1,650 A W^−1^ at 633 nm. The facile patterning, integration and packaging of high-quality two-dimensional chalcogenide crystals hold promise for innovations of next-generation photodetector arrays, wearable electronics and integrated optoelectronic circuits.

## Abstract (web summary)

Two-dimensional chalcogenides offer great potential in electronics, but accurate control of their growth is difficult. Here, the authors combine microintaglio printing and van der Waals epitaxy to pattern various large-area arrays of single-crystal chalcogenides with remarkable properties.

## Graphical abstract

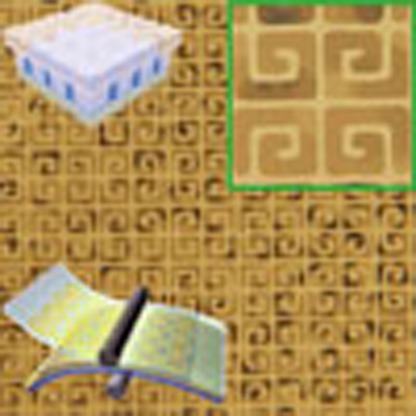

Two-dimensional (2D) chalcogenide crystals are single- and few-layer chalcogenides with a unique planar structure, such as 2D transition-metal chalcogenides NbSe_2_ and MoS_2_, III–VI In_2_Se_3_ and GaSe, IV–VI SnSe and V–VI Bi_2_X_3_ (X=Se or Te). This big family offers a potentially high mobility and rich electronic band structures ranging from semimetals to semiconductors and even topological insulators, having emerged as attractive candidates for the applications in electronics and optoelectronics^1^,^2^,^3^,^4^,^5^,^6^,^7^,^8^,^9^,^10^. In this family, layered Bi_2_X_3_ were recently discovered as topological insulators, a new state of quantum matter with an insulating bulk state and gapless Dirac-type edge/surface states^11^,^12^,^13^. Compared with bulk materials, 2D crystals of Bi_2_X_3_ topological insulators with large surface-to-volume ratios and enhanced exotic surface states provide excellent 2D geometry for the fabrication and integration of functional devices^3^,^14^,^15^. In_2_Se_3_, another intriguing chalcogenide was recently synthesized in atomically thin flakes. Transistors based on 2D In_2_Se_3_ crystals showed an efficient photoresponsivity, made them promising candidate in high-performance optoelectronics^16^.

However, to achieve the batch production, fabrication and integration of 2D chalcogenide crystals into electronic and optoelectronic devices, it is particularly important to pattern single crystals with minimal cross-talks^17^,^18^. A key requirement is the control of crystallization, specifically, the complete control of single-crystalline domain nucleation and growth, which is essential to the large-area patterning of high-quality 2D crystals^18^,^19^,^20^.

Herein, by combining microintaglio printing with van der Waals epitaxy, we have developed a method to efficiently pattern various 2D chalcogenide crystals onto mica substrates. Large-area arrays of 2D single-crystal chalcogenides with remarkable properties can be readily prepared. We believe that this patterning method paves the way for the application of 2D chalcogenide crystals in electronics and optoelectronics.

## Results

### Patterning of 2D chalcogenide crystals

**Figure 1 f1:**
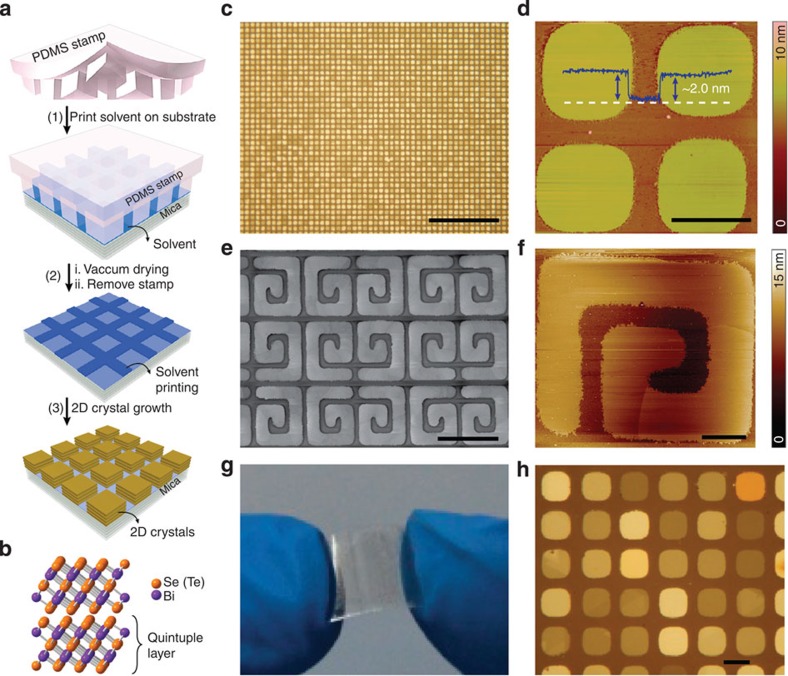
Patterning of 2D chalcogenide crystals. (**a**) Schematic representation of procedures for patterning of 2D chalcogenide crystals. (**b**) Layered crystal structure of chalcogenide topological insulators Bi_2_X_3_ (X=Se, Te), which consist of QLs. (**c**) Typical reflection-mode OM image of large-area 2D Bi_2_Se_3_ crystal array patterned on mica substrate. Scale bar, 200 μm. (**d**) AFM image of a 2 × 2 Bi_2_Se_3_ crystal array with a uniform thickness of 2 nm. Scale bar, 10 μm. (**e**) Scanning electron microscopy image of an array of spiral Bi_2_Se_3_ crystals. Scale bar, 50 μm. (**f**) AFM image of a spiral Bi_2_Se_3_ crystal. Scale bar, 10 μm. (**g**) Photograph of a mica substrate after patterning of 2D Bi_2_Se_3_ crystals that contains more than 200,000 crystals. (**h**) Typical reflection-mode OM image of 2D In_2_Se_3_ crystal array patterned on mica substrate. Scale bar, 10 μm.

Figure 1a illustrates the patterning process of 2D chalcogenide crystals on layered pseudohexagonal fluorophlogopite mica [KMg_3_(AlSi_3_O_10_)F_2_] substrates. As an ideal van der Waals epitaxy substrate^21^, freshly cleaved mica surface was first intaglio printed by solvent ink using a polydimethylsiloxane (PDMS) stamp, which has a surface relief structure containing the desired patterns. After microintaglio printing and vacuum drying of solvent ink, the mica surface modified with intaglio patterns was released from the PDMS stamp and used for selective-area van der Waals epitaxy of various 2D chalcogenide crystals in a set-up similar to our previous nanoribbon and nanoplate synthesis (see Methods, Supplementary Fig. 1, Supplementary Fig. 2 and Supplementary Note 1 for a detailed description)^8^,^14^. The pattern of as-grown various 2D crystals is faithfully reproduced from the relief pattern of the PDMS stamp.

The crystal structure of layered Bi_2_X_3_ is schematically depicted in Fig. 1b. Each covalently bonded X–Bi–X–Bi–X quintuple layer (QL) has a unit thickness of ∼1 nm, which assembles into 2D crystals mediated by van der Waals interactions. Figure 1c shows a typical optical microscopy (OM) image of a patterned 2D Bi_2_Se_3_ crystal array on a mica substrate. This array is comprised of about 2,000 discrete square nanoplates with an identical feature size—a side length of ∼10 μm and a periodicity of ∼15 μm—which renders a density of 1,700 nanoplates per inch. Importantly, the uniform crystallization and layer-by-layer growth mode facilitate the thickness control of 2D crystals (Supplementary Fig. 3 and Supplementary Note 2). As a result, 2D Bi_2_Se_3_ crystal arrays with domains of ∼10 μm in size and a uniform thickness of as thin as ∼2 nm have been achieved (Fig. 1d, Supplementary Fig. 4 and Supplementary Note 3). Besides discrete square nanoplates, more complex 2D crystal arrays with different feature sizes and pattern layouts have been created by using special PDMS stamps with desired patterns (Supplementary Fig. 5). An example of spiral arrays of 2D Bi_2_Se_3_ nanoplates and the corresponding atomic force microscopy (AFM) image are shown in Fig. 1e,f, [Fig f1], respectively. The terrace structures with multiple single QL steps along the spiral direction of 2D Bi_2_Se_3_ crystals were observed (Fig. 1f), which is reminiscent of the layer-by-layer growth mode. Furthermore, a large-area array of Bi_2_Se_3_ crystals containing more than 200,000 discrete crystals was successfully grown on a 1 × 1 cm flexible transparent mica substrate (Fig. 1g), which can be further scaled up for the batch production by repeatedly using intaglio printing on a larger mica substrate.

In addition to the facile patterning of 2D Bi_2_Se_3_ crystals, this method can be applied to the patterning of a broad range of 2D chalcogenide crystals. Examples of 2D chalcogenide crystal arrays of III–VI In_2_Se_3_ and GaSe, IV–VI SnSe and V–VI Bi_2_Te_3_ patterned on mica surfaces are shown in Supplementary Fig. 6. Among them, layered In_2_Se_3_ is an intriguing optoelectronic semiconductor with direct bandgap of ∼1.3 eV although In_2_Se_3_ is chemically and structurally compatible to topological insulator Bi_2_Se_3_ (ref. 16). Ordered arrays of 2D In_2_Se_3_ crystals with hexagonal layered phase have been also patterned on mica using this approach (Fig. 1h, Supplementary Fig. 7).

### Mechanism of patterning 2D chalcogenide crystals

**Figure 2:**
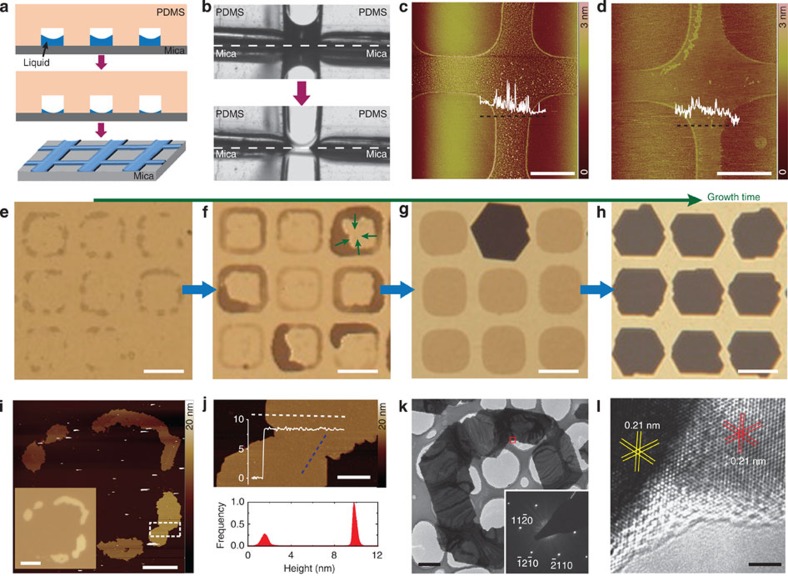
Mechanism of patterning 2D chalcogenide crystals. (**a**) Schematic of microintaglio printing process to form imprints on mica surface. (**b**) Charge-coupled device camera photos showing the transfer of solvent ink from a microwell of PDMS stamp onto mica. (**c**,**d**) AFM images of the imprints on mica surface captured directly after microintaglio printing (**c**) and after heating at 700 °C for 1 h (**d**). Scale bars in **c** and **d**, 5 μm. (**e–h**) Transmission-mode OM images of Bi_2_Se_3_ crystals on mica at different nucleation and growth stages: the nucleation of Bi_2_Se_3_ crystal islands starting from the edge of the imprints (**e**), epitaxial growth of Bi_2_Se_3_ crystal ring from the edge to the middle (**f**), formation of complete patterns after merging of different islands (**g**), the formation of thicker, single crystals at longer growth time (**h**). Scale bars in **e**–**h,** 10 μm. (**i**) AFM image of a patterned Bi_2_Se_3_ crystal in the middle stage of the growth process. Scale bar, 3 μm. Inset of **i**: reflection-mode OM image of the same crystal. Scale bar of **i** inset, 5 μm. (**j**) Enlarged AFM image of the merging region of two domains indicated in the box of **i**. Scale bar, 500 nm. Below panel of **j**: height distribution diagram of the indicated area. (**k**) TEM image of a patterned Bi_2_Se_3_ crystal in the middle stage of the growth process. Scale bar, 2 μm. Inset of **k**: diffraction pattern of the common merged area indicated by a red square showing single-crystal nature. (**l**) HRTEM image from the merging region indicated in **k**. Scale bar, 2 nm.

Understanding the crystal growth mechanism of 2D arrays is related to how to precisely control the patterning process and is therefore critical to achieve high device uniformity and high device yield for the practical applications in the large-area nanoelectronics and optoelectronics. To this end, we systematically investigated the intaglio printing process, nucleation and growth of 2D crystals. The solvent ink is usually made from the PDMS oligomer, which is dissolved in the most common organic solvent such as acetone, ethanol and cyclohexane. During the intaglio printing, the solvent ink is transferred onto the mica surface from microwells or cells of the patterned PDMS stamp (Fig. 2a). We *in situ* observed the transfer process of the solvent ink between the PDMS stamp and the mica surface using a contact angle goniometer (Fig. 2b). Interestingly, the ink surface inside the wells of PMDS forms a concave meniscus due to the capillary action and wettability during the solvent evaporation (Supplementary Table 1), which gives rise to the additional aggregation of residue along the edge of imprint patterns. After drying of the solvent ink, imprints are formed on the non-contacted regions between the PDMS stamp and mica surface. AFM measurements show that the imprints of PDMS oligomer have an average thickness of ∼0.5 nm and an root mean squared roughness of ∼0.4 nm at room temperature (Fig. 2c). After 1 h heating at 700 °C, the imprints can still be seen by AFM (Fig. 2d). In a controlled experiment, an aqueous solution ink of NaCl was used to form a heat-stabilized inorganic crystal imprint on mica, which further confirms the intaglio printing process (Supplementary Fig. 8 and Supplementary Note 4).

We find that imprints of solvent intaglio-printed onto mica provide the control over the nucleation of vapour-grown 2D chalcogenide crystals on the contacted regions of mica. Interestingly, the additional aggregation of residue at the edge of the imprints facilitates the nucleation of 2D seeds (Fig. 2e). As the growth continues, 2D seeds grown up from different nuclei can coalesce to form large-size seed aggregates, rings, and eventually continuous nanoplates on mica (Fig. 2f,g). Thicker nanoplates were obtained in the layer-by-layer growth mode by varying the growth time and deposition temperature (Fig. 2h). Figure 2i,j shows typical AFM images of merged areas at which adjacent islands coalesced together. To further examine the microstructure of merged areas by transmission electron microscopy (TEM), as-grown discrete 2D crystals were faithfully transferred from the mica substrate to a lacey carbon support film on a TEM grid using an efficient poly(methyl methacrylate)-mediated transfer technique (Supplementary Fig. 9, Supplementary Fig. 10 and Supplementary Note 5)^22^. Importantly, extensive TEM studies indicated that these discrete 2D crystal islands with six-fold symmetry are unidirectionally aligned and then merge to uniform single-crystal nanoplate rings with predefined orientation and smooth surface (Fig. 2k,l, Supplementary Fig. 10 and Supplementary Note 6). Note no discontinuous lines or wrinkles were observed in the merged areas. Eventually, 2D-ordered arrays of continuous nanoplates without any noticeable grain boundary defects were formed on prepatterned mica. Besides the identical orientation of multiple islands, both the ultraflat mica surface (root mean squared roughness ∼0.07 nm, Supplementary Fig. 11) and weak interlayer interaction facilitate seamless, defect-free coalescence of the adjacent layered islands during the van Waals epitaxy.

### Evaluation of patterned 2D chalcogenide arrays

**Figure 3:**
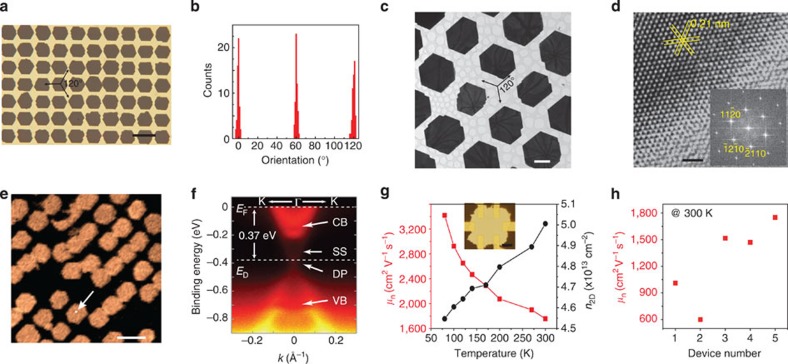
Evaluation of the crystal structure and electronic structure of 2D chalcogenide crystal arrays. (**a**) Typical transmission-mode OM image of 7 × 10 Bi_2_Se_3_ nanoplate array with the same orientation on a transparent mica substrate. Scale bar, 20 μm. (**b**) Histogram of the orientation distribution of Bi_2_Se_3_ nanoplates in **a**. (**c**) TEM image of a patterned Bi_2_Se_3_ crystal array transferred to a lacey carbon support film on a TEM copper grid. Scale bar, 5 μm. (**d**) HRTEM image obtained from the same Bi_2_Se_3_ crystal array, with corresponding fast Fourier transformation pattern shown in the inset. Scale bar, 1 nm. (**e**) Full valance spectra contrast map of a 2D Bi_2_Se_3_ crystal array measured by micro-ARPES with beam spot size of <1 μm and a 1-μm step scan. Scale bar, 20 μm. (**f**) Electronic band structure of individual Bi_2_Se_3_ crystals, showing a direct bulk gap and surface states consisting of a single Dirac cone, a hallmark of the topological insulator. The conduction band (CB), valence band (VB), surface state (SS) and Dirac point (DP) are indicated, together with the Fermi energy (*E*_F_), the energy level of Dirac point (*E*_D_) and wave vector *k*. (**g**) Hall mobility (*μ*_n_) and carrier density (*n*_2D_) as a function of temperature for an individual Bi_2_Se_3_ crystal with the thickness of ∼30 nm. Inset: OM image of a Hall-bar device fabricated on an individual Bi_2_Se_3_ crystal from a 2D array. Scale bar of inset, 5 μm. (**h**) Statistic distribution of room temperature mobility of five devices based on the 2D Bi_2_Se_3_ crystal array, indicating high mobility and reasonable uniformity of 2D crystals.

We have systematically evaluated the crystal structure and electronic structure of as-grown 2D chalcogenide crystals. In the case of Bi_2_Se_3_, a well-ordered array of truncated triangular Bi_2_Se_3_ nanoplates was patterned on the mica surface, in which the edges of nanoplates on mica were oriented predominantly at multiples of∼60° (Fig. 3a,b), consistent with the 2D hexagonal lattice of the Bi_2_Se_3_ QL. TEM and selected area electron diffraction further indicate the excellent alignment and the epitaxial nature of 2D-ordered nanoplate arrays patterned on mica. HRTEM (high-resolution TEM) and corresponding fast Fourier transformation pattern of individual Bi_2_Se_3_ nanoplates reveal that nanoplates are single-crystalline rhombohedral phase with atomically smooth edges parallel to the (11–20) direction (Fig. 3c,d). Energy-dispersive X-ray spectroscopy analyses revealed uniform chemical composition along the entire plane with a Bi/X atomic ratio of 2:3 (Supplementary Fig. 12). In addition to highly crystalline structure, the electronic band structure of the individual 2D Bi_2_Se_3_ crystal was directly measured by microspot angle-resolved photoemission spectroscopy (micro-ARPES) with high spatial resolution (Fig. 3e,f, Supplementary Fig. 13 and Supplementary Note 7), which unambiguously reveals clear linear dispersion of the topological surface state, characteristic of the topological insulator^11^.

The high crystalline quality and robust topological surface states of 2D Bi_2_Se_3_ crystals allow us to explore their high mobility transport. We first measured the temperature-dependent carrier concentration and carrier mobility of a Hall bar device of a ∼30-QL-thick Bi_2_Se_3_ nanoplate directly fabricated by electron beam lithography (EBL) on mica substrates (Fig. 3g, Supplementary Fig. 14 and Supplementary Note 8). It was observed that the carrier concentration decreases and the carrier mobility increases with decreasing temperature from 300 K down to 80 K, showing a semiconductor-like behaviour. Remarkably, a room temperature carrier mobility *μ*_n_=1,750 cm^2^ V^−1^ s^−1^ is obtained, which is the highest value reported for Bi_2_Se_3_ nanostructures such as nanowires^23^, nanoribbons^14^, 2D nanoplates^15^,^24^ and thin films^25^. Statistical measurements of carrier mobility indicate that our Bi_2_Se_3_ crystals have high mobility and reasonable uniformity (Fig. 3h and Supplementary Fig. 15). We speculate that the ultrahigh mobility is attributed to the suppression of scattering at grain boundaries and inter-terrace steps with improved quality of 2D crystals^25^,^26^.

### Optoelectronic measurements of patterned 2D crystals

**Figure 4:**
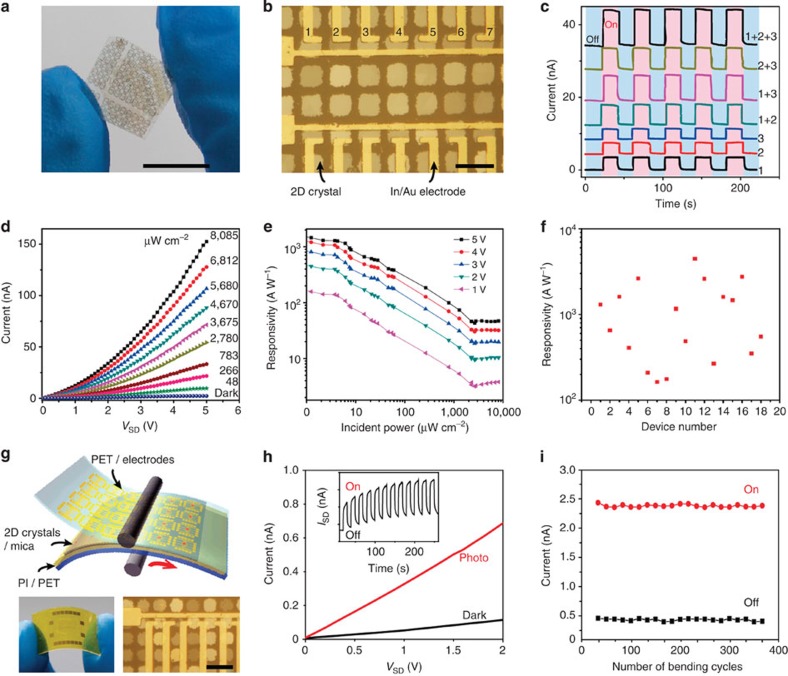
Electrical characterization, photodetector array and its packaged devices of patterned 2D crystals. (**a**,**b**) Photograph and OM image of In_2_Se_3_ photodetector arrays on the transparent and flexible mica substrate. Seven channels were labelled from 1 to 7, defined by the same source. Scale bar in **a**, 1 cm. Scale bar in **b**, 20 μm. (**c**) Photoresponse of multiple In_2_Se_3_ photodetector channels. Source–drain voltage (*V*_SD_)=0.1 V, light power density: 4 mW cm^−2^. (**d**) Current–voltage (*I*_SD_–*V*_SD_) curves of the individual device in the dark and under different illumination intensities with a 633-nm defocused laser. (**e**) Dependence of photoresponse on different illumination intensities and bias voltage. The device exhibits a photoresponsivity of 1,650 A W^−1^ under bias of 5 V for an illumination power intensity of 622 nW cm^−2^ and a device area of 40 μm^2^. (**f**) Statistic distribution of photoresponsivity of 18 devices based on the semiconducting In_2_Se_3_ crystal array on mica substrates. (**g**) Schematic of device packaging, the photograph of packaged device and its OM image. Electrodes and In_2_Se_3_ were pressed together by a lamination process. Electrodes (Ti/Au, 10 nm/40 nm) were defined onto the PET films by photolithography and thermal evaporation. Scale bar, 20 μm. (**h**) Photoresponse characterization of the packaged device. Inset: time-dependent photocurrent response of the packaged In_2_Se_3_ device at a bias voltage of 5 V. (**i**) Current as a function of bending cycles (before/after bending to a 10 mm radius) of the packaged In_2_Se_3_ devices on mica protected by PET with the laser on or off at a bias voltage of 5 V.

The excellent transport properties of 2D chalcogenide crystals facilitate high-performance optoelectronic applications because of their sizable bandgaps, large surface-to-volume ratio and the absence of dangling bonds^2^. Large-area, high crystalline quality nanoplates of 2D crystals grown on transparent dielectric mica substrates can provide a platform for transfer-free fabrication and measurements of flexible optoelectronic devices, such as transparent electrodes and photodetectors^8^. Grid networks containing 2D Bi_2_Se_3_ crystals with metallic surface states synthesized by this patterning technique are presented in Supplementary Fig. 16 and Supplementary Note 9, which can be used as novel broadband transparent flexible electrodes. On the other hand, the patterned In_2_Se_3_ crystal is *n*-type semiconductor with suitable direct bandgap (∼1.3 eV) and extraordinary photoresponse (Supplementary Fig. 17 and Supplementary Note 10). For practical high-performance photodetector applications, flexible In_2_Se_3_ photodetector arrays with several individual channels were directly fabricated by photolithography on the transparent mica substrate (Fig. 4a,b). Photocurrent (*I*_ph_) signals from individual channels as well as their additions were collected by illuminating the single and multiple channel devices (Fig. 4c, Supplementary Fig. 18), indicating realizable, stable, cooperative operations of high spatial resolution photodetector arrays.

## Discussion

One of the most striking properties of 2D chalcogenide crystals is their high photosensitivity, where the responsivity is the photocurrent *I*_ph_ divided by the incident power: *R*_ph_=*I*_ph_/*P*_in_. To gain further insight into the characteristics of the 2D photodetector, we extracted the representative *I*_SD_−*V*_SD_ output curves (Fig. 4d) and calculated the photoresponsivity (Fig. 4e) of 2D In_2_Se_3_ crystals illuminated at 633 nm under different incident light intensities. As the light intensity increases, more excited photocarriers are produced by interband transition and transported to the electrodes under the fixed bias, leading to more current. The photoresponsivity presents a non-linear dependence on the increasing incident light intensity and a final saturation behaviour, which may be attributed to the defects and/or charged impurities on the surface of the channel materials. This phenomenon has also been observed in 2D MoS_2_ photodetectors^27^. Under low incident power (*P*_in_=622 nW cm^−2^, 40 μm^2^), the In_2_Se_3_ device shows a remarkable responsivity of 1,650 AW^−1^ at 5 V bias, 10^5^ times higher than graphene-based photodetector (*R*_ph_=0.1∼10 mAW^−1^) and approximately two times higher than the best 2D MoS_2_ photodetector (*R*_ph_=880 AW^−1^)^4^. From the three-dimensional photoresponsivity map of In_2_Se_3_ photodetector (Supplementary Fig. 19), the photoresponsivity can be modulated by the bias voltage *V*_SD_, suggesting higher responsivity can be readily achieved by applying a larger bias voltage. Spatial photocurrent mapping of 2D In_2_Se_3_ crystals array shows a reasonable uniformity of the patterned crystal array (Supplementary Fig. 18), and the statistic measurements of 18 semiconducting In_2_Se_3_ photodetectors indicate 2D In_2_Se_3_ crystal arrays have high photoresponsivity with a reasonable uniformity (Fig. 4f). The photoresponsivity can range from several hundred to several thousand A/W, depending on the sample thickness, bias, incident light power and so on. The highest photoresponsivity of 2D In_2_Se_3_ crystal is measured as 4,470 A/W at a bias of 5 V (Supplementary Fig. 20).

The packaging is another important aspect for practical applications of optoelectronic devices. We exploited a lamination-based packaging method of patterned 2D chalcogenide crystals on mica^28^. Figure 4g shows a schematic diagram, representative photograph and OM image of packaged 2D optoelectronic devices. The device typically consists of three parts—a transparent polyethylene terephthalate (PET) previously coated with thin metal electrodes, a transparent flexible mica flake that supports 2D crystal array and a transparent PET substrate coated with a polyimide adhesive layer. When the three portions are put together to form a sandwiched configuration by OM-assisted physical lamination, van der Waals interactions between 2D crystals and metal electrodes facilitate the formation of intimate contact with clean interfaces, which may avoid the degeneration of devices introduced by standard lithography process and metal evaporation. This packaged photodetector exhibits a remarkable photoresponse (Fig. 4h) after switching incident light on or off many times. In addition, we investigated the mechanical durability of packaged 2D crystal photodetectors after repeated bending. Figure 4i shows the current as a function of bending cycles with the laser on or off at a bias voltage of 5 V. The packaged 2D In_2_Se_3_ photodetector kept its structural integrity, with little variation in both photocurrent and dark current after repeated dynamic bending tests, respectively, indicating its excellent flexibility and durability.

In summary, we have developed an efficient method to pattern high crystalline quality 2D chalcogenide crystals on mica substrates by the combination of microintaglio printing with van der Waals epitaxy. Accurate control of the crystallization, thickness, position, orientation and layout of patterned 2D chalcogenide crystals has been achieved. Large arrays of 2D single-crystal Bi_2_Se_3_ topological insulator with a record high Hall mobility show promise as broadband transparency flexible transparent electrodes. Furthermore, patterned 2D In_2_Se_3_ crystal arrays with an extraordinary photoresponsivity were integrated and packaged to flexible photodetectors. This facile patterning of high-quality 2D chalcogenide crystals would pave the route for next-generation photodetector arrays, wearable electronic and optoelectronic systems.

## Methods

### PDMS stamp fabrication

Relief structures consisting of desired patterns were fabricated in PDMS (Sylgard 184, Dow Corning) stamps using standard photolithography and replica moudling^29^. One layer of photoresist (diluted SU-8 2050, Microchem) with a thickness of ∼10 μm was first patterned on a SiO_2_/Si wafer using a Cr/quartz photomask to produce a master with a positive relief pattern. The master was cured for 20 min at 200 °C and then cooled down to room temperature. A PDMS stamp was cast onto the master by a mixture of the PDMS precursor and cross-linker (10:1 by mass), cured for 1 h at ∼90 °C and peeled away. For pre-extraction of PDMS oligomers, the PDMS stamp was allowed to swell in hexanes for 24 h. After drying for 0.5 h, the stamp was ultrasonicated three times in 2:1 ethanol–water mixture for 5 min. The overall pre-extraction process was repeated for three times to remove low molecular weight oligomers^30^.

### Microintaglio printing

Solvent ink (0.5–1 ml) such as acetone, ethanol or cyclohexane containing saturated PDMS oligomers was first pipetted onto the surface of PDMS stamps with microwell arrays. The array of microwells on the PDMS stamp filled with solvent ink was gently placed well-side down on the surface of freshly cleaved mica sheet, which were put in a vacuum chamber connected to a dry pump for about 5 min to dry organic solvent. The microintaglio-printed mica substrate was then removed from the PDMS stamp and immediately used for 2D chalcogenide crystal growth. We also developed a simple and fast method as an alternative to achieve the microintaglio printing of mica. A PDMS stamp without pre-extraction of oligomers was used to fill with solvent (acetone or ethanol) and gently press on the mica. The diffusion of PDMS oligomers into solvents act as no longer harmful contaminants but an effective ‘ink' for the microintaglio printing^30^.

### 2D chalcogenide crystal growth

We performed the growth of 2D chalcogenide crystals inside a 12-inch horizontal tube furnace (Lindberg/Blue M) equipped with a 1-inch diameter quartz tube. Chalcogenide powder (Alfa Aesar, Purity 99.999%) was placed in the hot center of the tube furnace as source material for evaporation and modified mica substrates were placed downstream 7–15 cm away from the source. Ultrapure argon was used as a carrier gas to transport the vapour to the colder furnace region. The tube was pumped to a base pressure of 50 mTorr and flushed with the carrier gas repeatedly to remove the oxygen residue. The van der Waals epitaxy growth of 2D chalcogenide crystals was performed under the conditions list in Supplementary Table 2.

### Hall bar device fabrication

Hall bar devices of individual 2D Bi_2_Se_3_ crystals on mica substrates were fabricated by EBL. First, as-patterned 2D crystals grown on mica substrate were predefined with alignment markers (10 nm Cr and 50 nm Au) made by photolithography and metal evaporation, followed by a second step of EBL to define multiple metal contacts of Hall-bar structures. Note that two layers of photoresist (conductive protective coating SX AR-PC-5000 and poly(methyl methacrylate)) were used to prevent the charge accumulation on the insulating mica substrate during EBL. The metal electrodes (5 nm Cr and 60 nm Au) were deposited by thermal evaporation, which were found to form nearly ohmic contact without annealing process. Silver paste was used to bond the devices for Hall measurements.

### Photodetector array fabrication

Standard photolithography was used to define the selected region and fabricate photodetector arrays of as-grown 2D chalcogenide crystals on mica substrates. The metal electrodes (10 nm In and 60 nm Au) were deposited by thermal evaporation, which were found to form ohmic contact to In_2_Se_3_ crystals (Supplementary Fig. 18). The devices were annealed at 300 °C for 30 min for avoiding the metallic phase. As for the packaging of flexible photodetector arrays, electrodes (10 nm Ti and 40 nm Au) were prefabricated on PET substrate by the photolithography and metal evaporation. Thin mica flakes patterned with 2D crystals were pasted onto another PET substrate precoated with a polyimide adhesive layer. These two portions were put together to form a sandwiched configuration by OM-assisted physical lamination, which conform the intimate lamination and optoelectronic measurements of packaged flexible device.

### Characterization

Characterization was done using OM (Olympus DX51 microscope), AFM (Vecco Nanoscope IIIa), scanning electron microscopy (Hitachi S-4800, acceleration voltage 5–30 kV) and TEM (FEI Tecnai F30, acceleration voltage 300 kV). Lacey carbon film supported on copper grids was used for TEM characterization. Micro-ARPES measurements were conducted at the spectromicroscopy beamline at Elettra Synchrotron Radiation lab in Italy, with energy resolution at 70 meV and angle resolution 0.5°. To avoid charging effects, samples were partially covered with aligned arrays of carbon nanotubes dry-transferred from the vertical array of carbon nanotube forests. The surface of the 2D crystal sample was cleaned by several cycles of Ar-sputtering and annealing under vacuum before the micro-ARPES measurements.

### Transport and photocurrent measurements

A semiconductor analyser (Keithley, SCS-4200) combined with a Micromanipulator 6,200 probe station was used to carry out the room temperature electrical measurements under ambient conditions. Magnetoresistance measurements on Hall bar samples were performed by a lock-in technique in a Quantum Design PPMS instrument, Janis-9T magnet He-cryostats and a Keithley S110 Hall effect measurement system. The temperature range is 2–300 K.

The photodetector devices were in series with fixed resistor (*R*) for the a.c. photovoltage measurement. Photocurrent (*V*_AC_/*R*) maps were achieved by a lock-in amplifier with a mechanical chopper (570 Hz) modulated laser and the Keithley 2,400 providing the bias voltages. A 632.8-nm Griot He–Ne laser was focused on samples through a Ni-U Nikon optical microscope with a micro-stage with alignment accuracy of better than 0.1 μm. The laser power was calibrated by an optical power metre (Newport, model 840-C). For the photocurrent characterization of the packaged device, the measurements were carried out under defocusing laser (633 nm) at a bias voltage 5 V.

## Author contributions

H.P. conceived and designed the experiments. W.Z., T.X. and Y.Z. performed the synthesis, structural characterization, device fabrication, transport measurements and analyses. Y.L.C. performed the micro-ARPES measurements. W.J., S.Z., J.Y., J.W., Y.J., Y.G., Y.W., G.C., S.H., H.Q.X. and Z.L. assisted in experimental work and contributed to the scientific discussions. W.Z., T.X., Y.Z. and H.P. wrote the paper. All the authors discussed the results and commented on the manuscript.

## Additional information

**How to cite this article:** Zheng, W. *et al*. Patterning two-dimensional chalcogenide crystals of Bi_2_Se_3_ and In_2_Se_3_ and efficient photodetectors. *Nat. Commun.* 6:6972 doi: 10.1038/ncomms7972 (2015).

## Supplementary Material

Supplementary Information Supplementary Figures 1-20, Supplementary Tables 1-2, Supplementary Notes 1-10 and Supplementary References

## References

[b1] Novoselov K. S. . Two-dimensional atomic crystals. Proc. Natl Acad. Sci. USA 102, 10451–10453 (2005). 16027370 10.1073/pnas.0502848102 PMC1180777

[b2] Wang Q. H., Kalantar-Zadeh K., Kis A., Coleman J. N. & Strano M. S. Electronics and optoelectronics of two-dimensional transition metal dichalcogenides. Nat. Nanotechnol. 7, 699–712 (2012). 23132225 10.1038/nnano.2012.193

[b3] Fiori G. . Electronics based on two-dimensional materials. Nat. Nanotechnol. 9, 768–779 (2014). 25286272 10.1038/nnano.2014.207

[b4] Koppens F. H. L. . Photodetectors based on graphene, other two-dimensional materials and hybrid systems. Nat. Nanotechnol. 9, 780–793 (2014). 25286273 10.1038/nnano.2014.215

[b5] Coleman J. N. . Two-dimensional nanosheets produced by liquid exfoliation of layered materials. Science 331, 568–571 (2011). 21292974 10.1126/science.1194975

[b6] Chhowalla M. . The chemistry of two-dimensional layered transition metal dichalcogenide nanosheets. Nat. Chem. 5, 263–275 (2013). 23511414 10.1038/nchem.1589

[b7] Huang X., Zeng Z. Y. & Zhang H. Metal dichalcogenide nanosheets: preparation, properties and applications. Chem. Soc. Rev. 42, 1934–1946 (2013). 23344899 10.1039/c2cs35387c

[b8] Peng H. L. . Topological insulator nanostructures for near-infrared transparent flexible electrodes. Nat. Chem. 4, 281–286 (2012). 22437712 10.1038/nchem.1277

[b9] Geim A. K. & Grigorieva I. V. Van der Waals heterostructures. Nature 499, 419–425 (2013). 23887427 10.1038/nature12385

[b10] Chang C.-Z. . Experimental observation of the quantum anomalous Hall effect in a magnetic topological insulator. Science 340, 167–170 (2013). 23493424 10.1126/science.1234414

[b11] Zhang H. J. . Topological insulators in Bi_2_Se_3_, Bi_2_Te_3_ and Sb_2_Te_3_ with a single Dirac cone on the surface. Nat. Phys. 5, 438–442 (2009).

[b12] Hasan M. Z. & Kane C. L. Colloquium: topological insulators. Rev. Mod. Phys. 82, 3045–3067 (2010).

[b13] Qi X.-L. & Zhang S.-C. Topological insulators and superconductors. Rev. Mod. Phys. 83, 1057–1110 (2011).

[b14] Peng H. L. . Aharonov-Bohm interference in topological insulator nanoribbons. Nat. Mater. 9, 225–229 (2010). 20010826 10.1038/nmat2609

[b15] Xiu F. X. . Manipulating surface states in topological insulator nanoribbons. Nat. Nanotechnol. 6, 216–221 (2011). 21317891 10.1038/nnano.2011.19

[b16] Lin M. . Controlled growth of atomically thin In_2_Se_3_ flakes by van der Waals epitaxy. J. Am. Chem. Soc. 135, 13274–13277 (2013). 23978251 10.1021/ja406351u

[b17] Aizenberg J., Muller D. A., Grazul J. L. & Hamann D. R. Direct fabrication of large micropatterned single crystals. Science 299, 1205–1208 (2003). 12595685 10.1126/science.1079204

[b18] Briseno A. L. . Patterning organic single-crystal transistor arrays. Nature 444, 913–917 (2006). 17167482 10.1038/nature05427

[b19] Aizenberg J., Black A. J. & Whitesides G. M. Control of crystal nucleation by patterned self-assembled monolayers. Nature 398, 495–498 (1999).

[b20] Minemawari H. . Inkjet printing of single-crystal films. Nature 475, 364–367 (2011). 21753752 10.1038/nature10313

[b21] Koma A. Van der Waals epitaxy for highly lattice-mismatched systems. J. Cryst. Growth 201, 236–241 (1999).

[b22] Jiao L. Y. . Creation of nanostructures with poly(methyl methacrylate)-mediated nanotransfer printing. J. Am. Chem. Soc. 130, 12612–12613 (2008). 18763767 10.1021/ja805070b

[b23] Hong S. S., Zhang Y., Cha J. J., Qi X.-L. & Cui Y. One-dimensional helical transport in topological insulator nanowire interferometers. Nano Lett. 14, 2815–2821 (2014). 24679125 10.1021/nl500822g

[b24] Yan Y., Wang L.-X., Yu D.-P. & Liao Z.-M. Large magnetoresistance in high mobility topological insulator Bi_2_Se_3_. Appl. Phys. Lett. 103, 033106 (2013).

[b25] Kou X. F. . Epitaxial growth of high mobility Bi_2_Se_3_ thin films on CdS. Appl. Phys. Lett. 98, 242102 (2011).

[b26] Najmaei S. . Vapour phase growth and grain boundary structure of molybdenum disulphide atomic layers. Nat. Mater. 12, 754–759 (2013). 23749265 10.1038/nmat3673

[b27] Lopez-Sanchez O., Lembke D., Kayci M., Radenovic A. & Kis A. Ultrasensitive photodetectors based on monolayer MoS_2_. Nat. Nanotechnol. 8, 497–501 (2013). 23748194 10.1038/nnano.2013.100

[b28] Lee T.-W., Zaumseil J., Bao Z., Hsu J. W. P. & Rogers J. A. Organic light-emitting diodes formed by soft contact lamination. Proc. Natl Acad. Sci. USA 101, 429–433 (2004). 14704283 10.1073/pnas.0304179101 PMC327164

[b29] Xia Y. N. & Whitesides G. M. Soft lithography. Angew. Chem. Int. Ed. 37, 550–575 (1998). 10.1002/(SICI)1521-3773(19980316)37:5<550::AID-ANIE550>3.0.CO;2-G 29711088

[b30] Lee J. N., Park C. & Whitesides G. M. Solvent compatibility of poly (dimethylsiloxane)-based microfluidic devices. Anal. Chem. 75, 6544–6554 (2003). 14640726 10.1021/ac0346712

