# Supplementary material for: Patterning two-dimensional chalcogenide crystals of Bi2Se3 and In2Se3 and efficient photodetectors
Source: Nat Commun. 2015 Apr 21;6:6972. doi: 10.1038/ncomms7972 (PMC4411293; doi:10.1038/ncomms7972)
Supplement: Supplementary Information — Supplementary Figures 1-20, Supplementary Tables 1-2, Supplementary Notes 1-10 and Supplementary References [file ncomms7972-s1.pdf]

Supplementary Figures

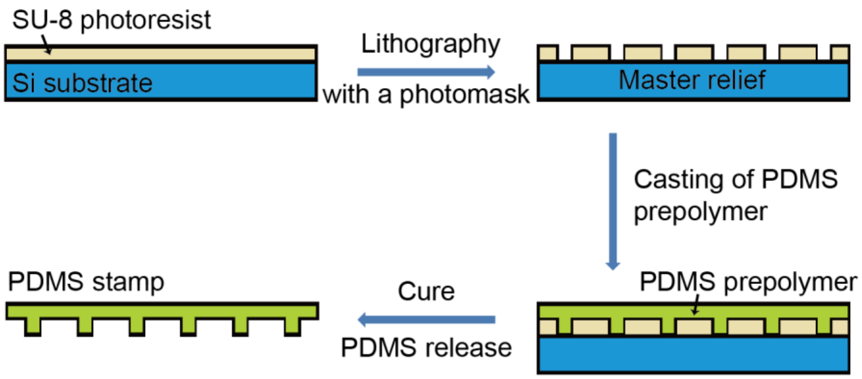

Supplementary Figure 1. Schematic of fabrication procedures of PDMS stamps and detailed procedure of PDMS stamp fabrication.

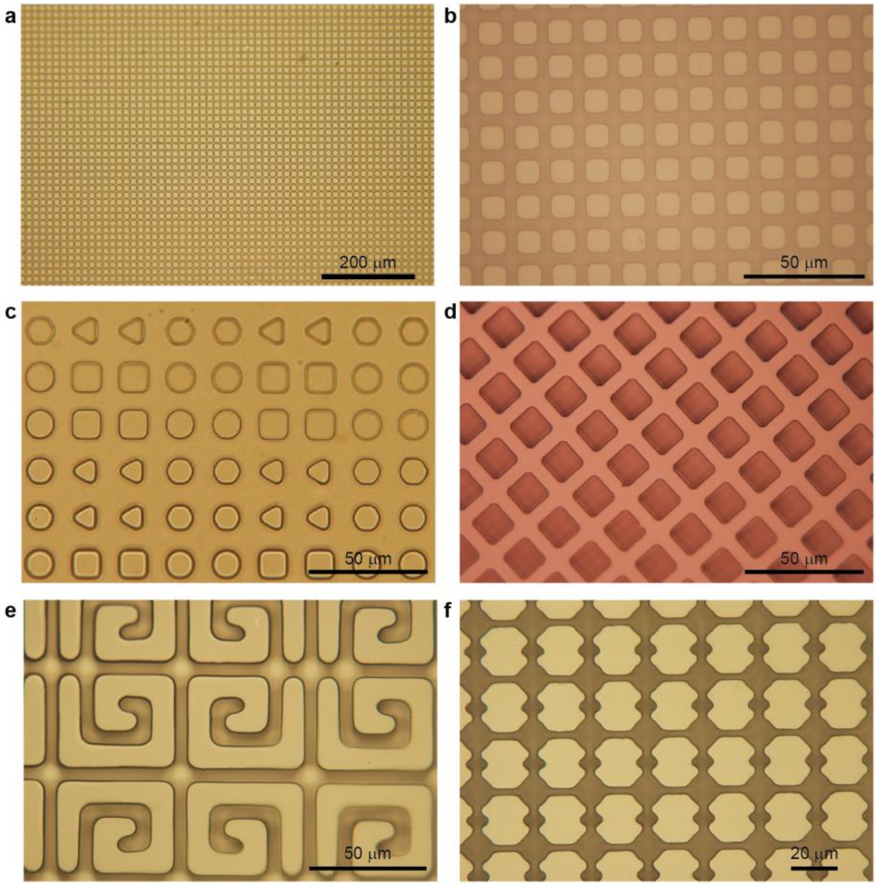

Supplementary Figure 2. OM images of typical PDMS stamps with a variety of relief structures. a, **b,** OM Images of PDMS stamps with square pillars which have a length of 10 μm and a periodicity of 15 μm. **c,** OM Image of a complex PDMS stamp with 2 × 2 hexagonal, triangular, circular and square pillars which have a periodicity of 20 μm. **d,** OM Image of a PDMS stamp with square wells which have a length of 15 μm and a periodicity of 20 μm. **e,** OM Image of a PDMS stamp with spiral patterns. **f,** OM Image of a PDMS stamp with dumbbell-like patterns.

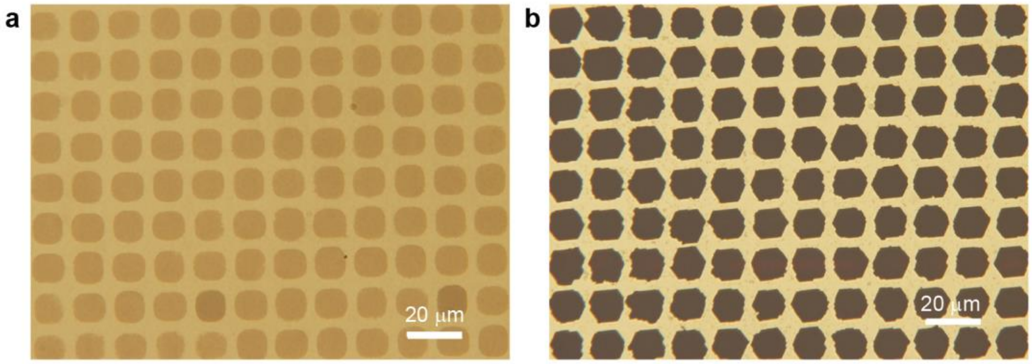

Supplementary Figure 3. Thickness control of 2D crystals. a, OM image of an array of ultrathin Bi_2Se_3 nanoplate with ~2 nm in thickness. **b,** OM image of an array of thick Bi_2Se_3 nanoplates with ~30 nm in thickness.

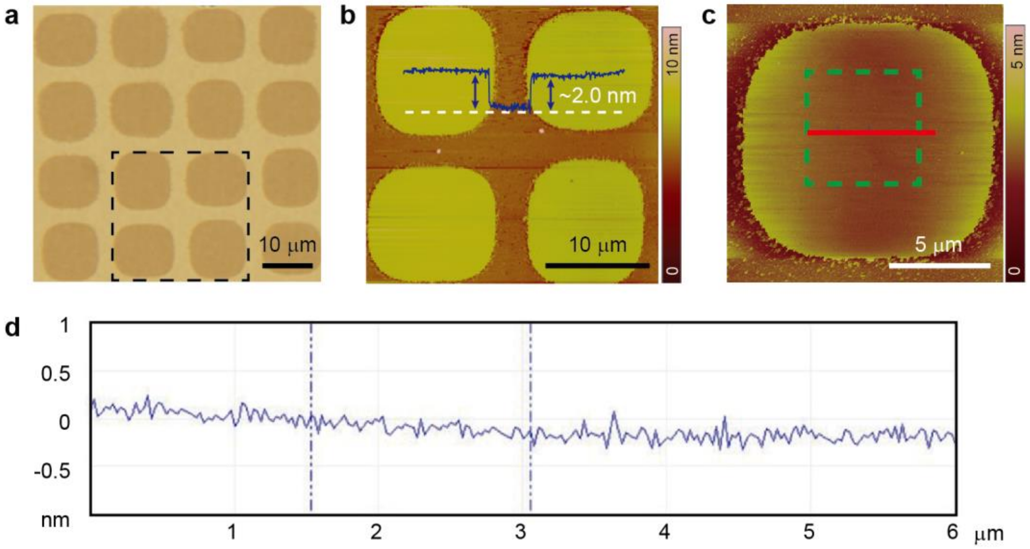

Supplementary Figure 4. Patterned arrays of ultrathin 2D Bi_2Se_3 crystals. **a**. OM image of an array of ultrathin 2D Bi_2Se_3 crystals with uniform thickness of ~2 nm. **b**. AFM image of the 2 × 2 Bi_2Se_3 nanoplate array shown in the dashed black box in (**a**). **c**. Detailed AFM image of the nanoplate in the left bottom corner in (**b**). **d**. Height profile along the red line in (**c**), confirming the ultra-flat surface morphology.

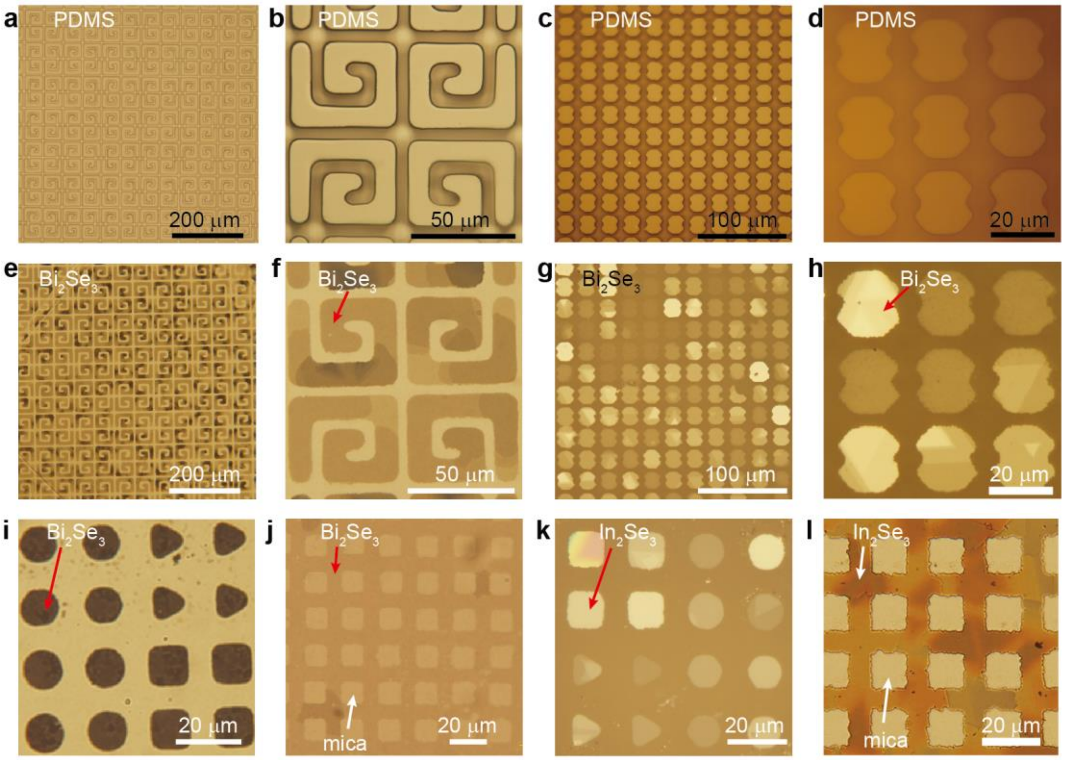

Supplementary Figure 5. Complex 2D crystal arrays with different feature sizes and pattern layouts grown on mica using special PDMS stamps with desired patterns. a-b, Reflection-mode OM images of PDMS consisting of spiral pattern arrays. **c-d,** Reflection-mode OM images of PDMS consisting of dumbbell-like pattern arrays. **e-f,** Transmission-mode OM images of spiral nanoplate arrays of Bi_2Se_3 patterned with the PDMS stamps shown in (a,b). **g-h,** Reflection-mode OM images of dumbbell-like nanoplate arrays of Bi_2Se_3 patterned with the PDMS stamps shown in (c,d). **i,** Transmission-mode OM images of patterned 2D Bi_2Se_3 with different shapes: 2×2 hexagonal (top left), triangular (top right), circular (bottom left) and square (bottom right) nanoplates patterned at nearby regions. The periodicity is 20 μm. **j,** Patterned 2D Bi_2Se_3 mesh with a periodicity of 20 μm. **k**, OM image of ultrathin patterned 2D In_2Se_3 with different structures at nearby regions. **l**, OM image of patterned 2D In_2Se_3 mesh.

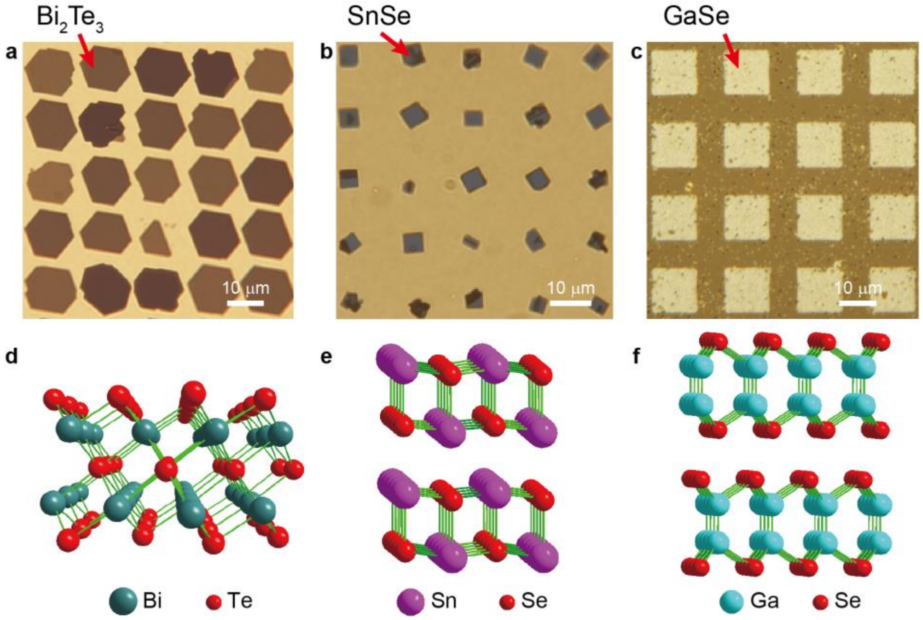

Supplementary Figure 6. Patterning of a variety of 2D chalcogenide crystals. a-c, OM images of patterned 2D V-VI chalcogenide Bi_2Te_3, IV-VI chalcogenide SnSe, and III-VI chalcogenide GaSe, respectively. **d-f,** the corresponding crystal structures of layered Bi_2Te_3, SnSe, and GaSe, respectively.

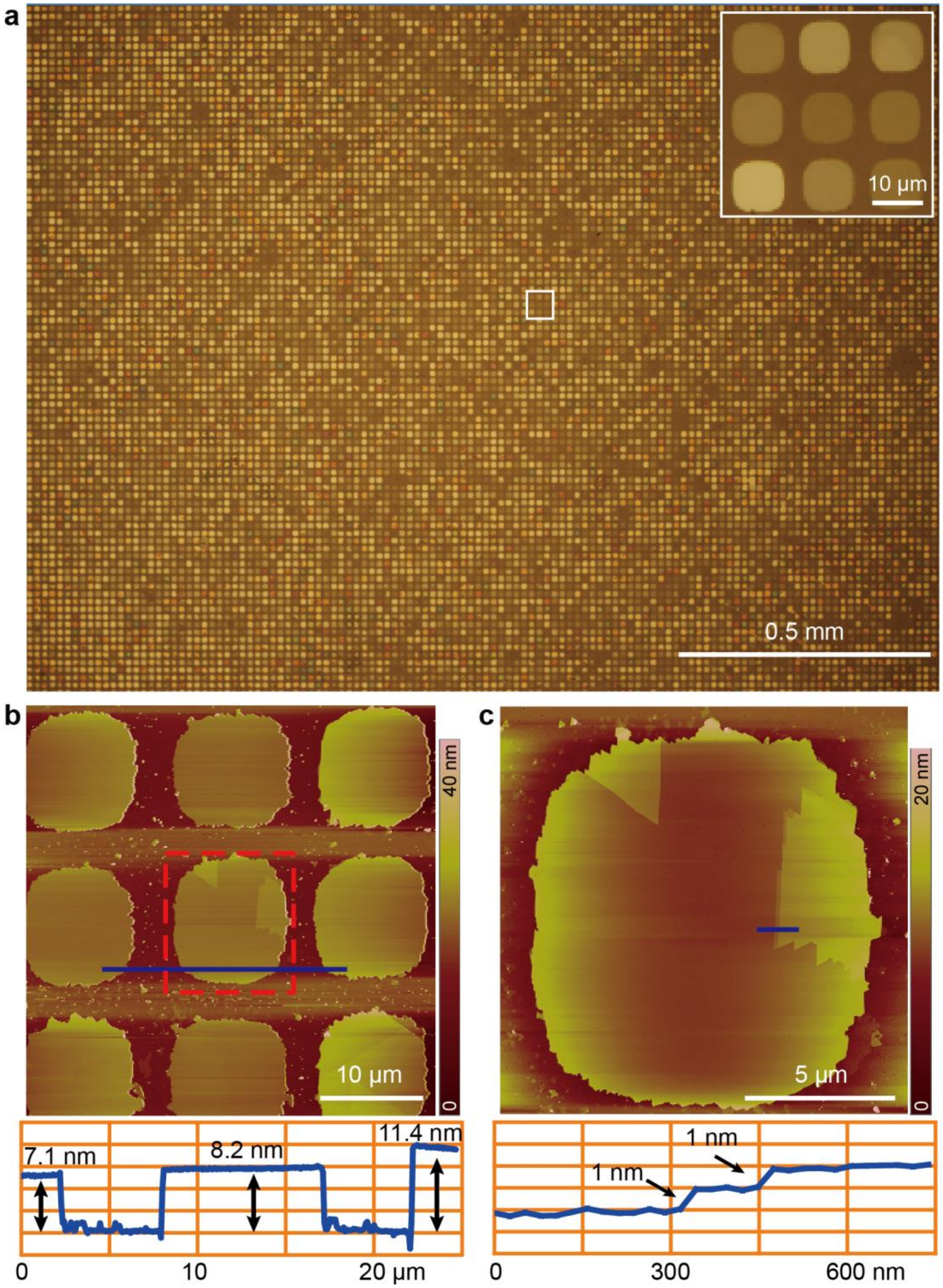

Supplementary Figure 7. OM and AFM images of patterned 2D In_2Se_3 crystal arrays. a, OM image of large area In_2Se_3 nanoplates patterned on transparent mica substrate. This 2.5 mm^2 region contains more than 10,000 uniform In_2Se_3 nanoplates. Inset: Enlarged image of 2D In_2Se_3 crystal array captured from the white box area of (a).

b, A 3 × 3 array of In_2Se_3 nanoplates and its corresponding height profile along the blue line in the AFM image. The thickness of In_2Se_3 nanoplates is around 8.2 nm, corresponding to 8 quintuple layers (QLs). **c,** Corresponding enlarged AFM image of the nanoplate shown in the dashed red box in (b). The height of the steps is about 1 nm which is consistent with the height of 1 QL of layered In_2Se_3.

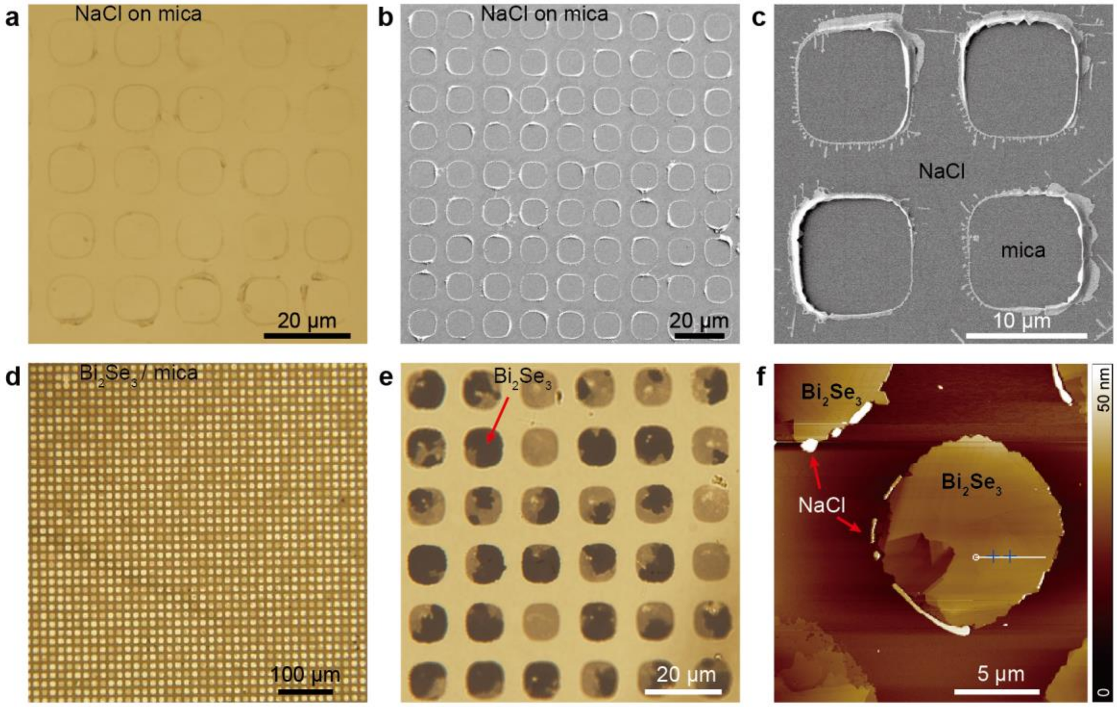

Supplementary Figure 8. Patterning of 2D Bi_2Se_3 crystals using NaCl aqueous solution as an ink. a, Transmission-mode OM image of NaCl crystal imprint on mica using 0.1 mol/L NaCl aqueous solution as the ink. **b-c,** Typical SEM images of NaCl crystal pattern micro-intaglio printed on mica. **d-e,** Transmission-mode OM images of 2D Bi_2Se_3 arrays grown on exposed mica surface using the NaCl crystal template. **f,** Typical AFM image of 2D layered Bi_2Se_3 crystals grown on the mica substrate defined by the 2D NaCl crystal template.

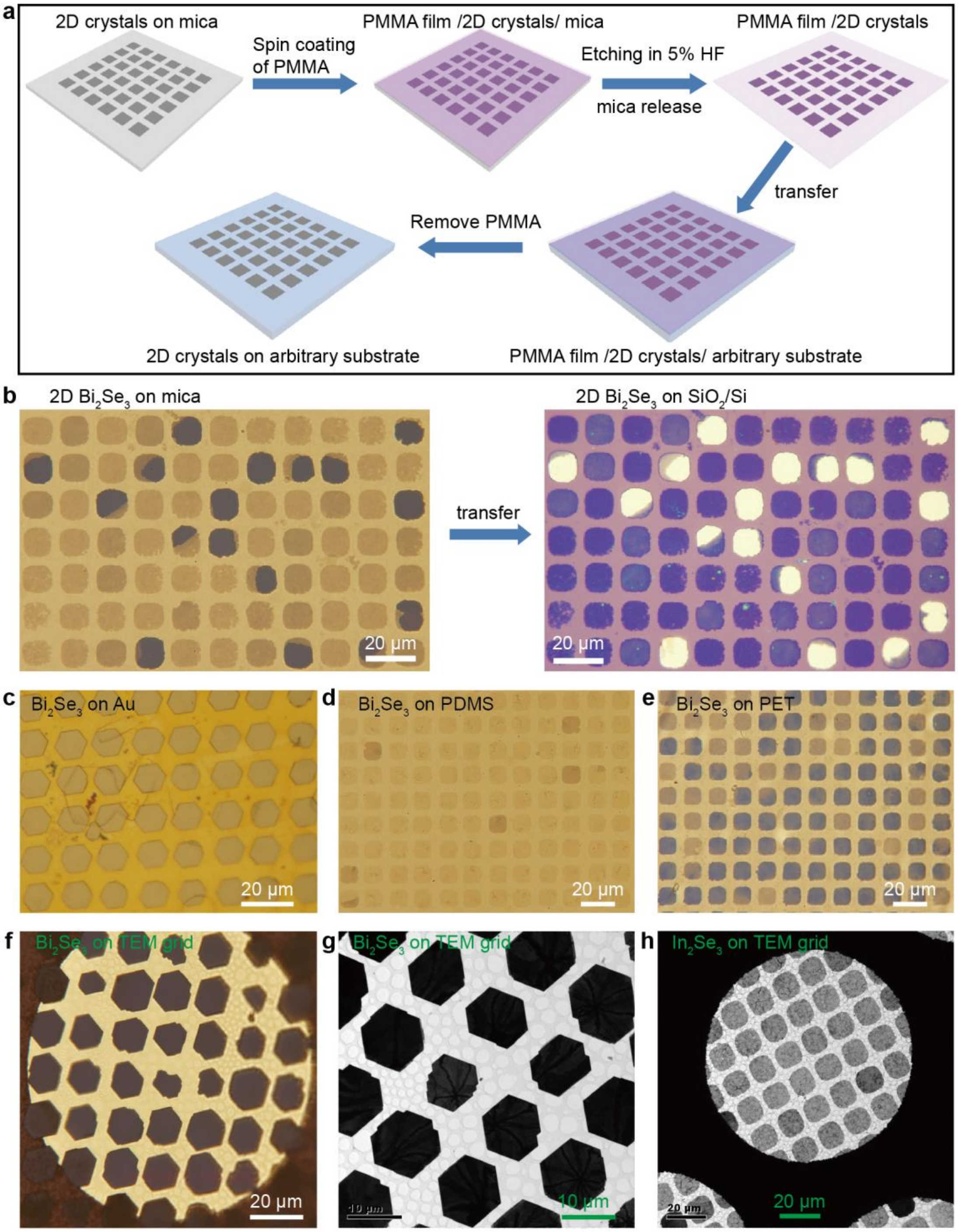

Supplementary Figure 9. Faithful transfer of 2D crystals from mica onto arbitrary substrates. a, Schematic diagram for the PMMA-mediated transfer technique.[2] **b,c,** Optical images of 2D Bi_2Se_3 crystals before and after transfer, respectively. **c-f**, Optical images of 2D Bi_2Se_3 crystals transferred onto thin Au film, a soft PDMS matrix,

transparent PET plastic, a TEM grid, respectively. **g**, TEM image of 2D Bi_2Se_3 crystals on a TEM grid. **h**, TEM image of 2D In_2Se_3 crystals transferred onto a TEM grid.

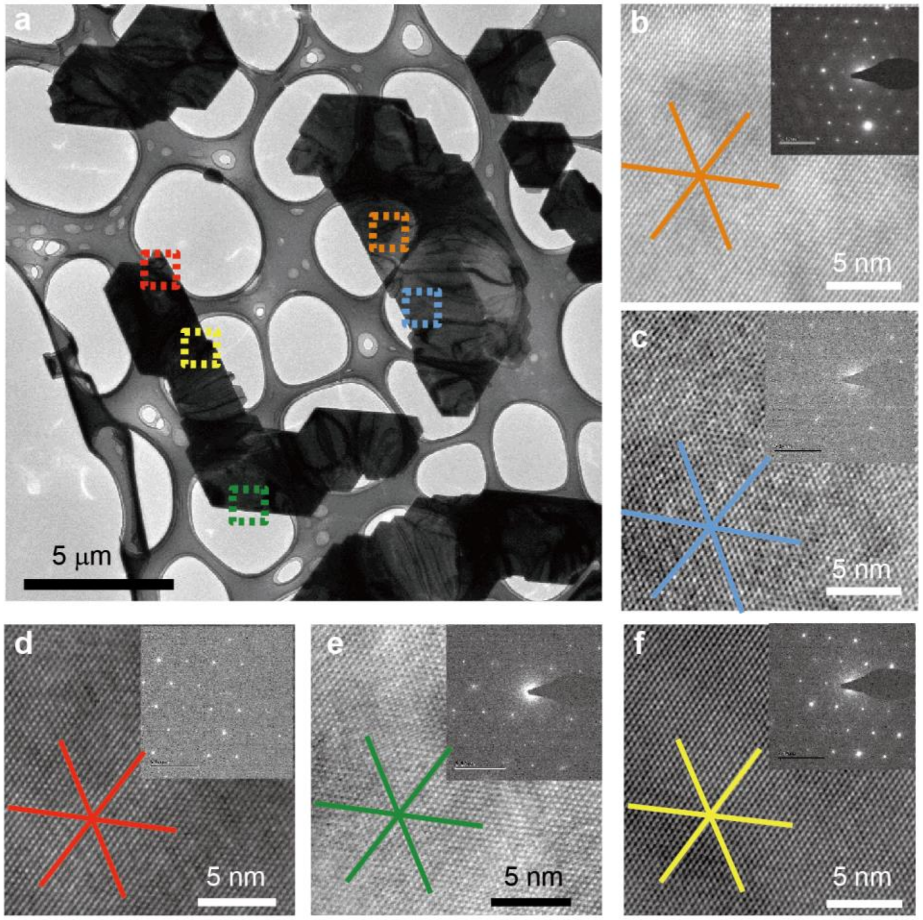

Supplementary Figure 10. Alignment and merging of 2D chalcogenide crystal islands. a, TEM image of 2D single crystals of Bi_2Se_3 that merged by several aligned crystal islands. **b-f,** HRTEM images and SAED patterns from selective areas indicated by color dash squares shown in (a). The orientation of crystal lattices and diffraction spots is identical, which indicates that 2D chalcogenide crystal islands can merge to a single crystal ring and nanoplate.

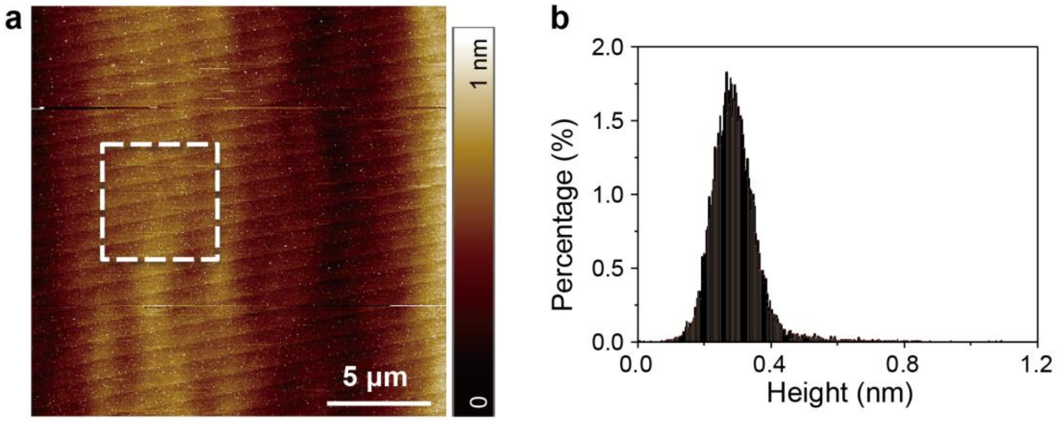

Supplementary Figure 11. Ultraflat surface of a mica substrate. a, Typical AFM image of the ultra-smooth surface of a freshly cleaved mica substrate. **b,** Height distribution diagram of indicated area shown in (**a**).

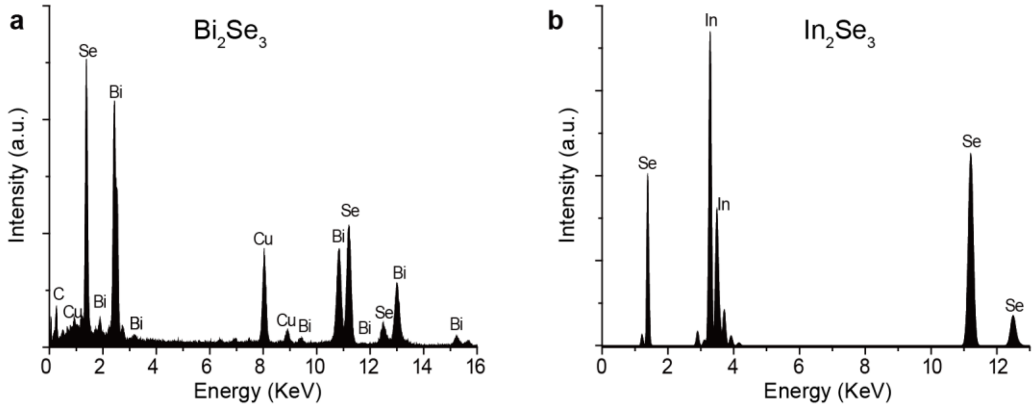

Supplementary Figure 12. EDX analyses of 2D crystals of Bi_2Se_3 (a) and In_2Se_3 (b). The Cu and C signals come from the TEM grid.

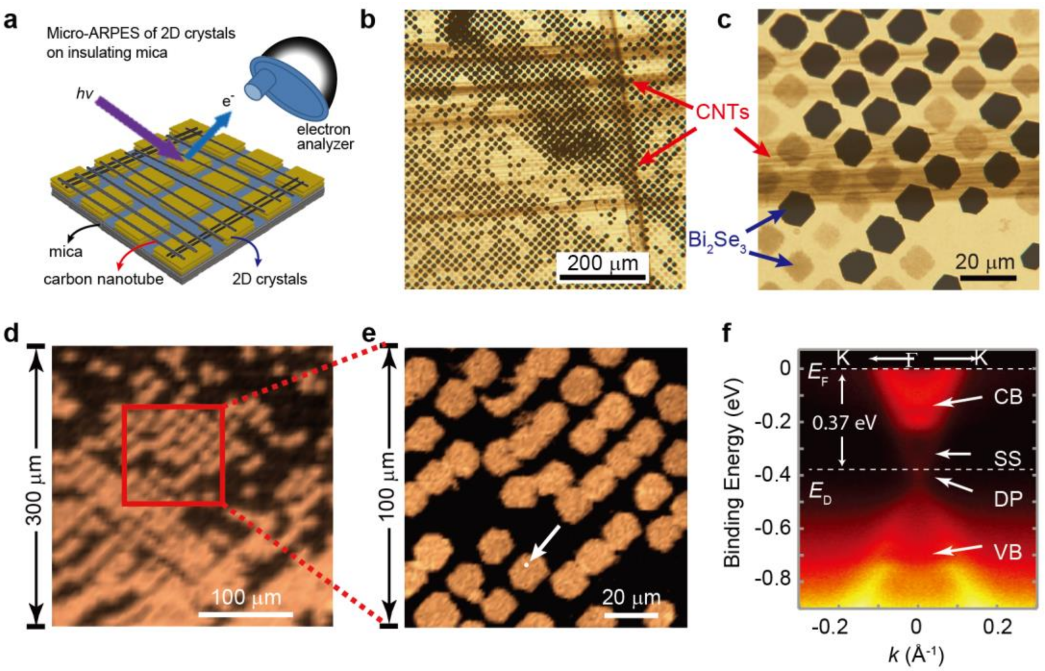

Supplementary Figure 13. Micro-ARPES measurements of Bi_2Se_3 crystal arrays grown on insulating mica substrates. a, Schematic of preparation of micro-ARPES samples to avoid charging effect of insulating mica substrate. The samples were covered with aligned arrays of carbon nanotubes dry-transferred from vertical array of carbon nanotube forests. **b,c,** Typical OM images of 2D Bi_2Se_3 crystal arrays grown on mica that were covered with aligned arrays of carbon nanotubes. **d,e,** Full valance spectra contrast maps of the 2D Bi_2Se_3 crystal array with coarse and fine scans, respectively. **f**, Measured electronic band structure of a Bi_2Se_3 crystal taken at the white arrow in (e) using micro-ARPES with beam spot size of <1 μ m, which reveals a direct bulk gap and surface states consisting of a single Dirac cone, a hallmark of the topological insulator. The conduction band (CB), valence band (VB), surface state (SS) and Dirac point (DP) are indicated, together with the Fermi energy (E_F), the energy level of Dirac point (E_D), and wave vector *k*.

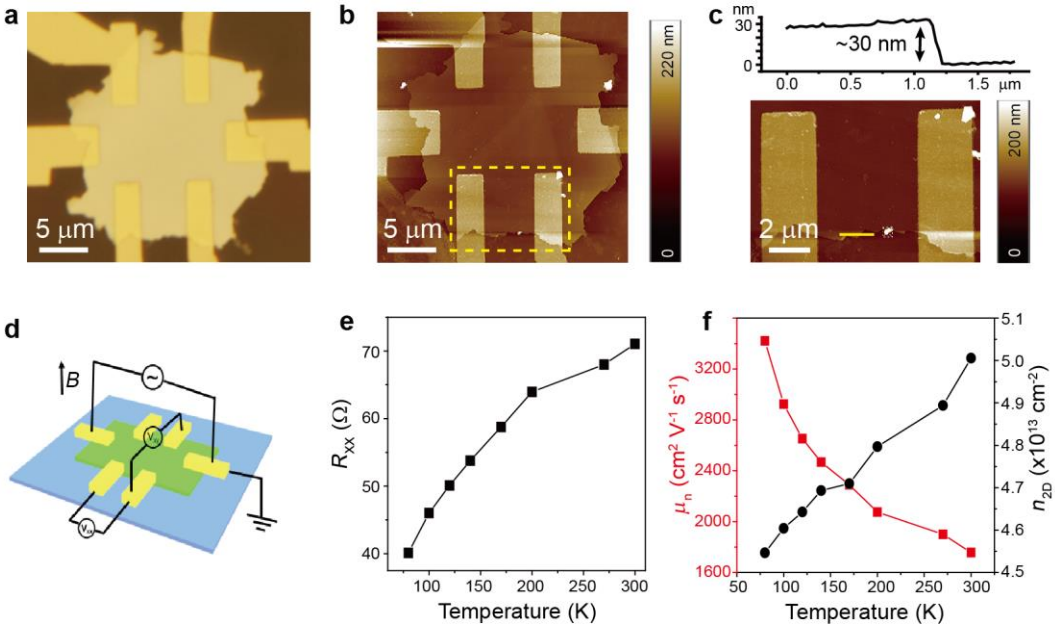

Supplementary Figure 14. Transport measurements of a ~30-QL-thick Bi_2Se_3 nanoplate. a, Typical optical microscopy image of a Hall-bar device fabricated on an individual Bi_2Se_3 crystal selected from a 2D array. **b,c,** AFM images of patterned grown Bi_2Se_3 Hall device and its height profile. **d,** The scheme of Hall measurements. **e,** The longitudinal resistance R_{XX} was plotted as a function of temperature. **f,** Hall mobility and carrier density as a function of temperature for an individual Bi_2Se_3 crystal.

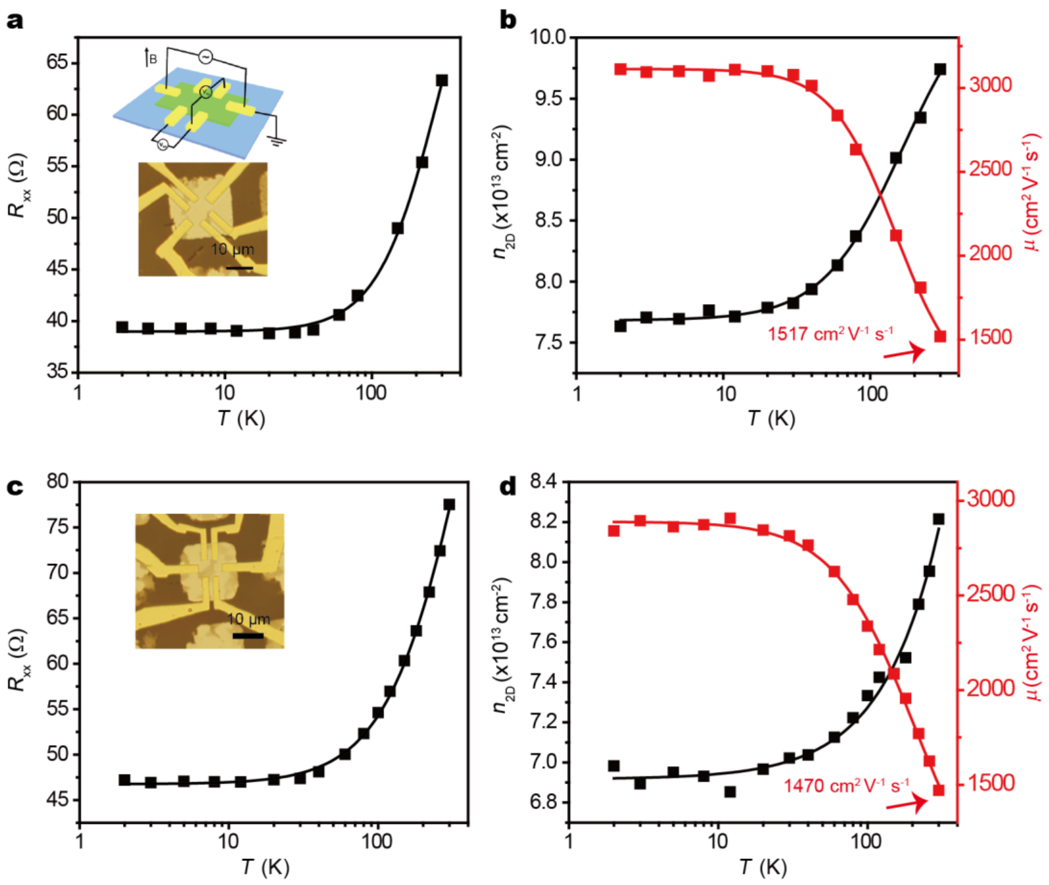

Supplementary Figure 15. Transport measurements of two Hall bar devices from the 2D Bi_2Se_3 crystal array. a, The longitudinal resistance R_{xx} was plotted as a function of temperature, inset: the scheme of Hall measurements and the optical image of 2D Bi_2Se_3 crystal. **b**, Hall mobility and carrier density as a function of temperature for the Bi_2Se_3 crystal device shown in (a). The room temperature mobility is 1517 cm^{2}V^{-1}s^{-1}. **c**, The longitudinal resistance R_{xx} was plotted as a function of temperature for a 2D Bi_2Se_3 crystal with a rough surface. **d**, Hall mobility and carrier density as a function of temperature for the Bi_2Se_3 crystal device shown in (c). The room temperature mobility is 1470 cm^{2}V^{-1}s^{-1}.

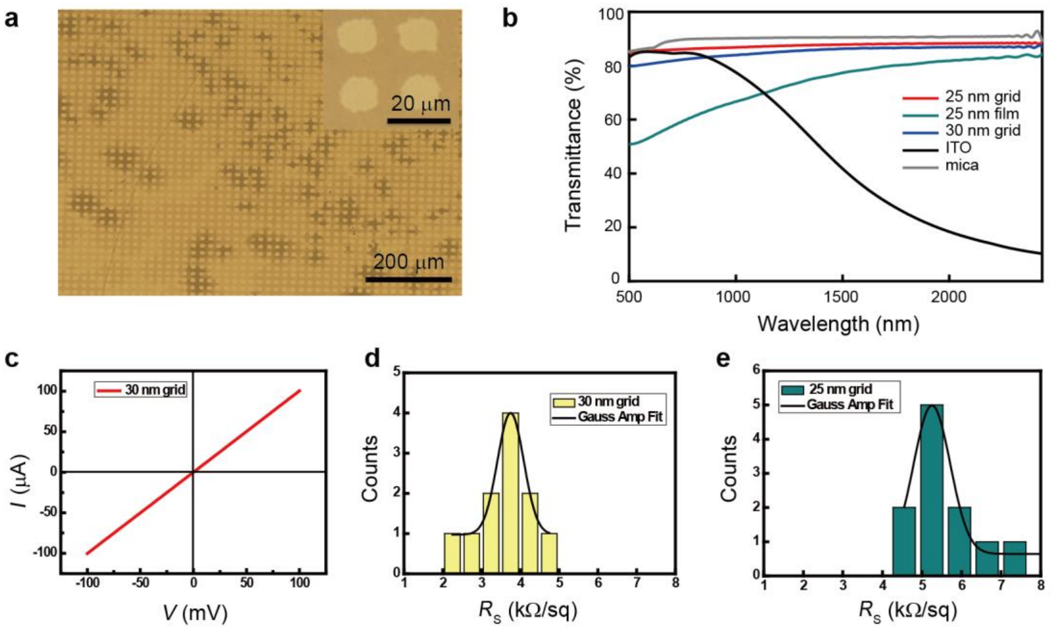

Supplementary Figure 16. Grid networks containing 2D Bi_2Se_3 crystals for flexible transparent electrodes. a, Typical optical microscopy images in transmission mode of large-area, few-layer Bi_2Se_3 grids with circular shaped apertures grown on mica substrates. **b,** Transmittance spectrum of Bi_2Se_3 grid electrodes with different thicknesses on mica, as well as for pure mica substrate (grey), ITO (black), and the 25-nm-thick Bi_2Se_3 film electrode (green). The sharp oscillatory features come from Fabry-Perot interference effects. **c,** Source-drain current (*I*) versus voltage (*V*) characteristics for Bi_2Se_3 grids. **d, e,** Histograms of sheet resistance distributions of Bi_2Se_3 grids with thickness of 30 nm and 25 nm, respectively.

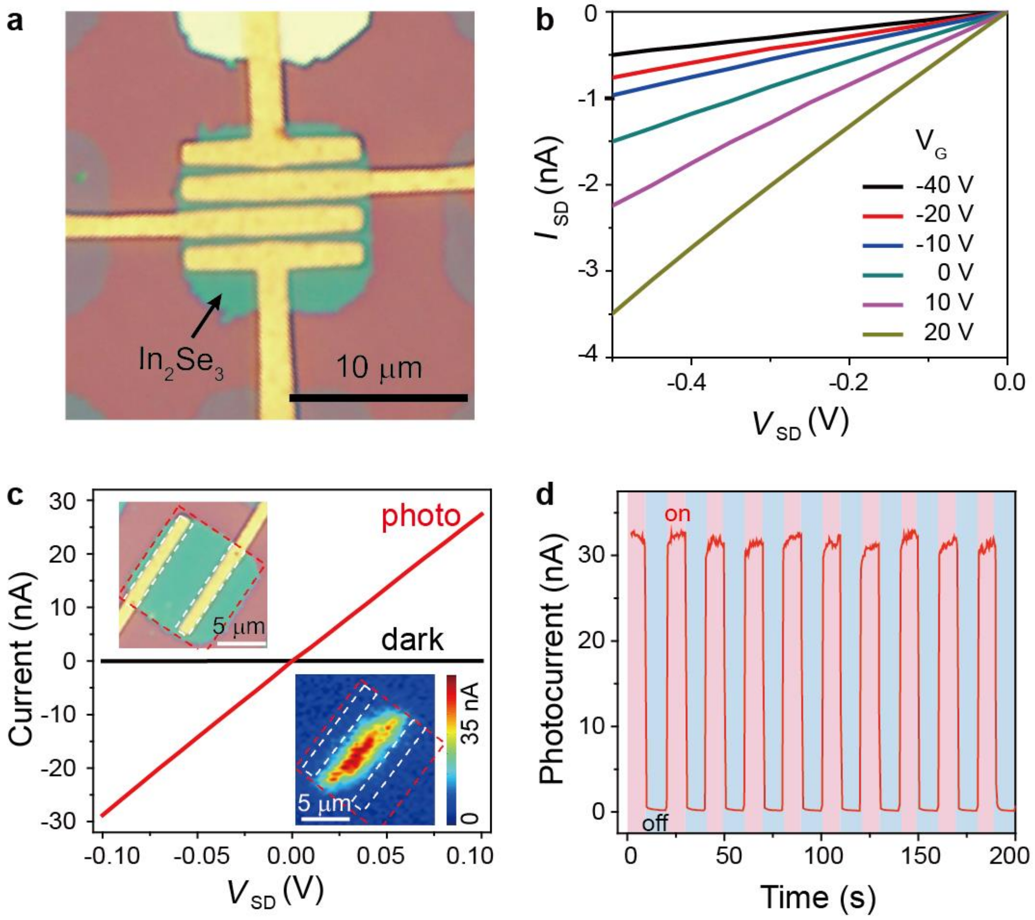

Supplementary Figure 17. Transport and photoresponse measurements of 2D In_2Se_3 crystals transferred onto SiO_2/Si substrate. a, Optical microscopy image of a back-gated FET device of indivudual In_2Se_3 crystal on SiO_2/Si substrate. **b,** Typical gate dependent dark current-voltage (I_{SD}-V_{SD}) curves of indivudual In_2Se_3 crystal at room temperature. **c,** Current versus source-drain bias with (red curve) and without (black curve) 633 nm laser beam focused at In_2Se_3 channel. V_g = 0 V, P_{inc} = 100 μW. Inset: Optical microscopy image of the device (left) and corresponding spatial photocurrent map (right) recorded by a focused laser beam scanning over the whole photodetector. Electrodes and In_2Se_3 channel indicated as dashed lines. V_{SD} = 1 V, V_g = 0 V, P_{inc} = 35 nW. **d,** Stable and repeatable photoresponse of the device with the light illumination switched off or on. V_g = 0 V, P_{inc} = 100 μW.

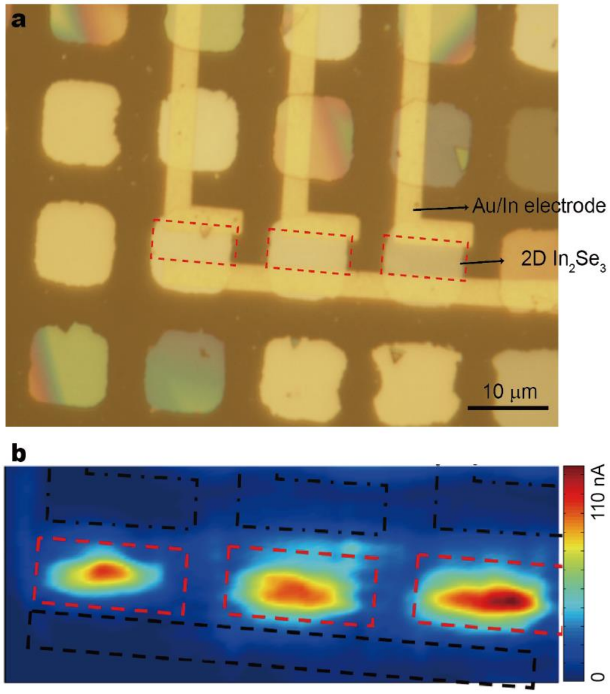

Supplementary Figure 18. Photocurrent mapping of a 2D In_2Se_3 crystal array based photodetection devices fabricated on mica substrate. a, Optical microscopy image of the photodetector array device. **b**, The spatial photocurrent map by a focused laser beam scanning over the whole photodetector array in a. Au/In electrodes and In_2Se_3 channels indicated as dashed lines. V_{sd} = 2 V, P_{inc} = 73 μW.

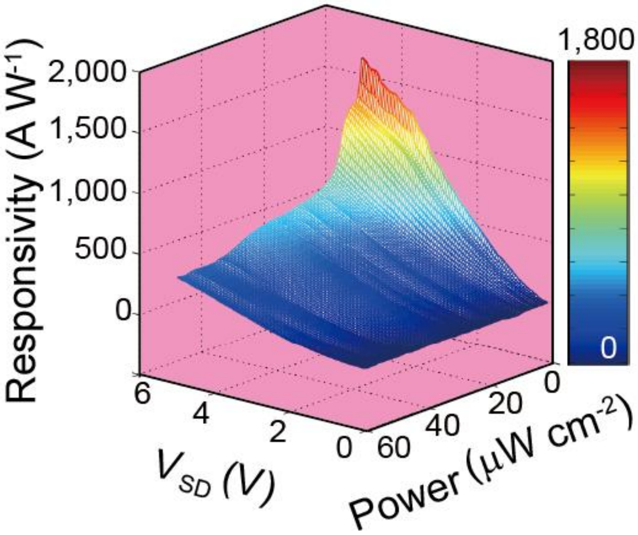

Supplementary Figure 19. 3D photoresponsivity map of the In_2Se_3 photodetector showing a remarkable responsivity of 1650 AW^{-1} at 5 V bias. The photoresponsivity can be modulated by the bias voltage, suggesting higher responsivity can be readily achieved by applying a larger bias voltage.

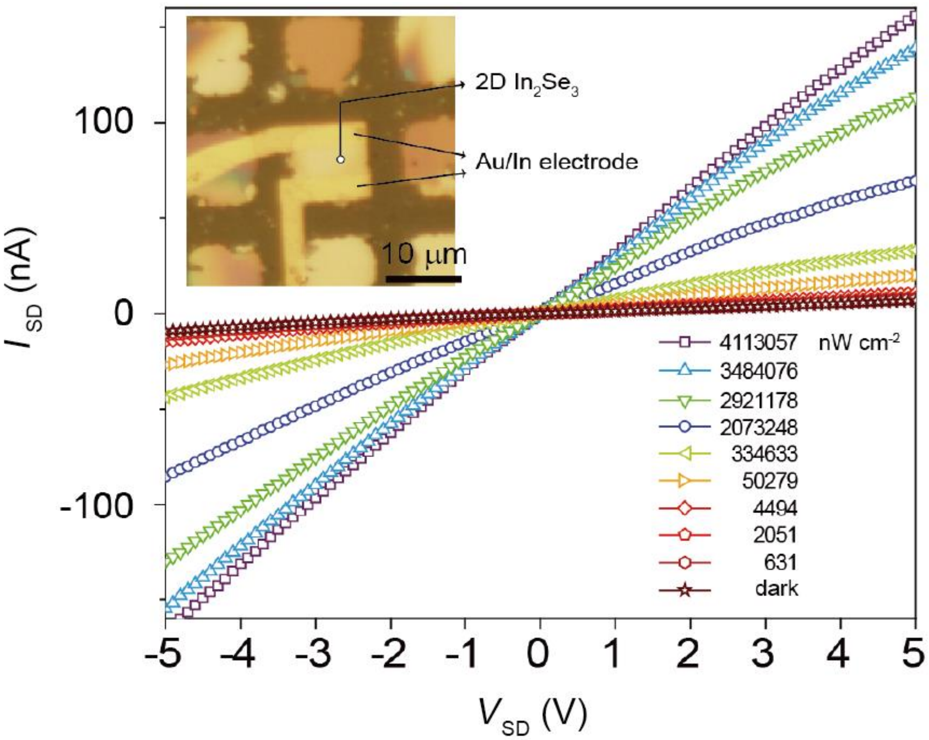

Supplementary Figure 20. The highest photoresponsivity of a 2D In_2Se_3 crystal grown on a mica substrate is measured as 4470 A/W at a bias of 5 V.

Supplementary Table 1. Wettability of PMDS stamp with different solvent

Solvent	Contact angle	Images obtained from contact angle goniometer
acetone	33.6 ± 2.0	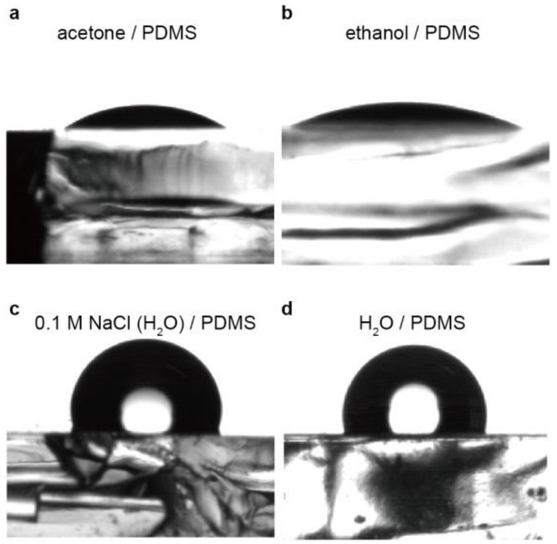
ethanol	25.6 ± 2.6	
NaCl in H_2O (0.1 mol/L)	106.4 ± 5.4	
H_2O	105.4 ± 1.7	

Supplementary Table 2. Growth conditions for various 2D crystals

	Source Temp. (ºC)	Substrate Temp. (ºC)	Flow rate (sccm)	Pressure (Torr)	Growth time (min)
GaSe	750-850	720-620	100	20	5-40
In_2Se_3	680-750	600-650	60-150	20-30	5-40
SnSe	550-650	380-500	100	40-60	5-40
Bi_2Se_3	480-530	330-430	80-500	20-100	5-500
Bi_2Te_3	450-500	300-400	100-300	20-100	5-60

Supplementary Note 1. Detailed procedure of PDMS stamp fabrication

Polydimethylsiloxane (PDMS) stamps with relief structures consisting of desired patterns were prepared with standard photolithography and replica molding technique (Supplementary Figure 1).[1] Photoresist SU-8 2050 was first mixed with cyclopentanone (25 % ~75 % volume ratio to SU-8 2050) to form diluted photoresist with similar properties (by mass percentage of solid) to SU-8 2010, SU-8 2007 or SU-8 2005. Then, the diluted SU-8 photoresist was patterned on a Si substrate using a chrome/quartz mask consisting of negative structures with standard photolithography and development process to produce a master with positive relief pattern. The master was cured at 200 ℃ for 20 min and then cooled down to room temperature to improve the interaction between the SU-8 photoresist and Si substrate. After that, a mixture of PDMS prepolymer and cross-linker (10:1 by mass) was poured on the master and cured at 90 ℃ for 1h. Finally, the PDMS mold was peeled off from the master and cut into 1.5 cm × 1.5 cm stamps. Supplementary Figure 2 shows some typical optical microscopy (OM) images of as-obtained PDMS stamps used for the micro-intaglio printing of solvent inks and the patterning of two-dimensional (2D) chalcogenide crystals.

Supplementary Note 2. Thickness control of 2D crystals

The uniform crystallization and layer-by-layer growth mode facilitate the thickness control of 2D crystals (Supplementary Figure 3). We controlled the thickness of 2D crystals primarily by changing the source temperature and growth time. Supplementary Figure 3 shows typical results of thickness controlled growth results of 2D Bi_2Se_3 nanoplate arrays. The only difference between the growth conditions for them is that the source temperature is 490 ℃ for Supplementary Figure 3a and 530 ℃ for Supplementary Figure 3b, respectively. If the growth time increases from 5 min that used for the sample in Supplementary Figure 3a to 8 hours, we can obtain a thick nanoplate array similar to the sample shown in Supplementary Figure 3b.

Supplementary Note 3. Ultrathin and uniform 2D crytals

By finely tuning the growth conditions and taking advantage of the uniform crystallization and layer-by-layer growth mode of 2D Bi_2Se_3 crystals, we can readily prepare 2D Bi_2Se_3 arrays with domains of ~10 μm in size and a uniform thickness of as thin as ~2 nm. (Fig. 1d, Supplementary Figure 4)

The r.m.s. roughness of the nanoplate surface shown in the green dashed square is as small as 0.091 nm, comparable to the roughness of freshly cleaved mica surface. This ultra-flat surface confirms the layer-by-layer growth mechanism.

Supplementary Note 4. Patterning of 2D crystals using an aqueous solution ink

We printed an aqueous solution ink of NaCl (~0.1 mol/L) to form an intaglio pattern containing heat-stabilized inorganic crystal imprint on mica using a PDMS stamp with relief structures (Supplementary Figure 8). This observation confirms the micro-intaglio printing process based on PDMS stamps. With the assistance of NaCl crystal template on mica, ordered array of 2D Bi_2Se_3 crystals was grown on exposed mica surface using the selective-area van der Waals epitaxy, which is faithfully reproduced from the relief structure of the PDMS stamp.

Supplementary Note 5. Faithful transfer of 2D crystals from mica onto arbitrary substrates

We have transferred the array of 2D chalcogenide crystals grown on mica substrates onto arbitrary substrates using a PMMA-mediated transfer technique.[2] First, a thin film of PMMA was formed to encapsulate 2D crystals on mica by spin-coating and baking of a PMMA solution (Micro Chem, 950 kg/mol, ~4 wt.% in anisole, spin-coating: 2000 rpm for 1 min, baking: 170 ^{o}C for 5 mins). A sticky tape with a square hole in the middle was pasted on the surface of PMMA/2D crystals/mica, then dip into HF solution (~5%) for about 30 mins to remove away the mica substrate. The 2D crystals/PMMA film supported by the sticky tape was transferred onto any arbitrary substrates. After the sticky tape was removed, the PMMA film was dissolved by acetone vapor, leaving the transferred 2D crystal array on the targeted substrate (Supplementary Figure 9a).

Supplementary Figure 9b and Supplementary Figure 9c show the optical images of 2D Bi_2Se_3 crystals with a variety of morphologies grown on a mica substrate and after transferred on a SiO_2/Si substrate, respectively, indicating an efficient and faithful transfer process. Supplementary Figure 9c-9f show typical transfer results of Bi_2Se_3 crystal arrays on thin Au film, a soft PDMS substrate, transparent PET plastic, and a TEM grid, respectively. Such PMMA-mediated transfer technique is quite facile preparation route to TEM samples of 2D crystals, convenient for investigation of microstructures by TEM (Supplementary Figure 9g and Supplementary Figure 9h).

Supplementary Note 6. Merging of aligned 2D crystal islands to a single crystal

Van der Waals epitaxy facilitates the growth of orientation-defined 2D crystals with six-fold symmetry on the surface of layered mica that has a pseudo-hexagonal structure. Due to the symmetry matching, 2D hexagonal crystals with identical orientations were predominantly grown on mica substrates. After transferring of 2D crystals onto the TEM grid, we observed that the orientations of the 2D nanoplates' edge were predominantly at multiples of ∼60°, consistent with the 2D hexagonal lattice. From the extensive HRTEM and SAED analyses, we found that the discrete 2D hexagonal crystal islands were unidirectionally aligned and then merged to uniform single-crystal nanoplate rings with predefined orientation and smooth surface (Supplementary Figure 10). The growth of orientation-defined 2D chalcogenide crystals on layered substrates reflects the essence of van der Waals epitaxy.

Supplementary Note 7. Micro-ARPES measurements of 2D Bi_2Se_3 crystal array on insulating mica

To directly observe the electronic band structure of individual 2D Bi_2Se_3 crystals, we have carried out micro-spot angle-resolved photoemission spectroscopy (micro-ARPES) on as-obtained Bi_2Se_3 crystal arrays grown on insulating mica substrates (Supplementary Figure 13). Micro-ARPES measurements were conducted at the spectromicroscopy beamline at Elettra Synchrotron Radiation lab in Italy, with the high spatial resolution of

below 1 μm, energy resolution at 70 meV and angle resolution 0.5 °. To avoid charging effect of mica substrates, samples were covered with aligned arrays of carbon nanotubes dry-transferred from vertical array of carbon nanotube forests. The exposed surface of the 2D crystal sample was cleaned by several cycles of Ar-sputtering and annealing under vacuum before the micro-ARPES measurements.

Supplementary Note 8. Transport measurements of Bi_2Se_3 nanoplates from a 2D crystal array

We selected individual 2D Bi_2Se_3 crystals from a crystal array grown on the mica substrate and directly fabricated a Hall-bar device by electron beam lithography (EBL). An additional conductive protective coating (SX AR-PC-5000) was used on PMMA photoresist to prevent the charge accumulation on the insulating mica substrate during EBL. The temperature-dependent carrier concentration and carrier mobility of a Hall bar device of a Bi_2Se_3 nanoplate was measured. The thickness of the Bi_2Se_3 nanoplate was determined by AFM to be ~ 30 QLs (Supplementary Figure 14). A room temperature carrier mobility μ_n = 1750 $cm^2 V^{-1} s^{-1}$ are obtained, which is the highest value reported for Bi_2Se_3 nanostructures such as nanowires, nanoribbons, 2D nanoplates, and thin films.

Supplementary Note 9. Grid networks containing 2D Bi_2Se_3 crystals for flexible transparent electrodes

Large-area and high-quality grid networks containing 2D Bi_2Se_3 crystals with controlled thickness and well-aligned positions are directly produced by this patterning technique on mica substrates (Supplementary Figure 16a). The 2D Bi_2Se_3 grid structures with metallic surface states can significantly improve the visible transparency in comparison with continuous films (Supplementary Figure 16b). As-grown topological grid electrodes exhibit a flat and high transmittance of more than 85 % from visible to near-infrared region. On the other hand, the sheet electrical conductivity of topological grid electrodes over large area can be as low as ~2.0 kΩ/sq. (Supplementary Figure 16c-16e) In addition to broadband transparence and good electrical conductivity, this high-

quality topological grid electrode possesses outstanding mechanical durability and excellent stability to environmental perturbations.

Supplementary Note 10. Transport and photoresponse measurements of 2D In_2Se_3 crystals transferred onto SiO_2/Si substrate

The patterned In_2Se_3 crystal is n-type semiconductor with suitable direct bandgap (~1.3 eV) and extraordinary photoresponse (Supplementary Figure 17). Electronic transport measurements on the 2D In_2Se_3 crystals were performed by using a back-gated FET configuration (Supplementary Figure 17a). The 2D In_2Se_3 crystals were transferred onto 300-nm-thick SiO_2/Si substrate and defined into four-probe configuration by standard electron beam lithography, followed by the thermal evaporation of metal contacts (10-nm-thick indium, 60-nm-thick gold). The typical electrical characterization of 2D In_2Se_3 crystal devices in dark state were shown in Supplementary Figure 17b. The twenty-three studied devices display similar behavior. The measured current-voltage curves at various gate voltages (V_g from -40 V to 20 V) are linear (Supplementary Figure 17b), indicating good ohmic contacts. The source-drain current increased when the gate voltage increased from -40 V to 20 V, which exhibit n-type nature of the In_2Se_3 transistor. As shown in Supplementary Figure 17c, the In_2Se_3 device produced the remarkable change of the slope of current-voltage curve when the channel illuminated by a focused laser, indicating that the photocurrent generation originated from the photoconductive mechanism. The spatial photocurrent map was recorded by a focused laser beam scanning over the whole photodetector under a fixed bias. It is distinct that the photocurrent was strongly generated in the whole 2D In_2Se_3 crystal channel, weakly in the two electrodes, which can attribute to the ohmic contacts between the In_2Se_3 channel and metallic electrodes. A stable and repeatable operation of dynamic photoresponse was observed in the 2D In_2Se_3 crystal. Supplementary Figure 17d presents the good photoresponse properties of the In_2Se_3 device with light illumination off and on. The photoresponse ratio (I_{light}/I_{dark}) is ~860.

Supplementary Reference

1. Xia, Y. & Whitesides, G. M. Soft lithography. ***Angew. Chem. Int. Ed.*** *37*, 550 (1998).
2. Jiao, L. Y.; Fan, B.; Xian, X. J.; Wu, Z. Y.; Zhang, J. & Liu, Z. F. Creation of nanostructures with poly(methyl methacrylate)-mediated nanotransfer printing**.** ***J. Am. Chem. Soc.*** *130*, 12612 (2008).
